# Supplementary material for: A spontaneous termination mechanism of RNA polymerase V shapes the DNA methylation landscape in plants
Source: EMBO J. 2026 Apr 2;45(9):3192–205. doi: 10.1038/s44318-026-00763-7 (PMC13144423; doi:10.1038/s44318-026-00763-7)
Supplement: Supplementary file 1 — Appendix [file 44318_2026_763_MOESM1_ESM.pdf]

Appendix for:

A spontaneous termination mechanism of RNA polymerase V shapes the DNA methylation landscape in plants

Table of Contents

Appendix Figure S1. A diagram of the experimental procedure of Pol V NET-seq and RIP-seq……………………………………………………………………………2
Appendix Figure S2. Statistics of the Pol V NET-seq and RIP-seq data…………..3
Appendix Figure S3. Arabidopsis Pol V transcription boundaries enrich T-rich sequence……………………………………………………………………………5
Appendix Figure S4. *In vitro* Pol V transcription assay with designed scaffolds…..7
Appendix Figure S5. Cryo-EM structure analysis of the 0U complex………………8
Appendix Figure S6. Cryo-EM structure analysis of the 1U complex……………..9
Appendix Figure S7. Cryo-EM structure analysis of the 2U complex……………10
Appendix Figure S8. Cryo-EM structure analysis of the 3U complex……………11
Appendix Figure S9. Cryo-EM structure analysis of the 4U complex……………12
Appendix Figure S10. Cryo-EM structure analysis of the 5U complex…………..13
Appendix Figure S11. Cryo-EM structure analysis of the 6U complex…………..14
Appendix Figure S12. Cryo-EM structure analysis of the 7U complex…………..15
Appendix Figure S13. Cryo-EM structure analysis of the 8U complex…………..16
Appendix Figure S14. The structures of 0U to 8U complexes and their comparison with the Pol V elongation conformation…………………………………………...17
Appendix Figure S15. Slow RNA Pol II of yeast (*S. pombe*) pauses at the T-tract..18
Appendix Table S1. Oligos used in this research…………………………………...19
Appendix Table S2. Cryo-EM data collection, refinement, and validation statistics…………………………………………………………………………....21

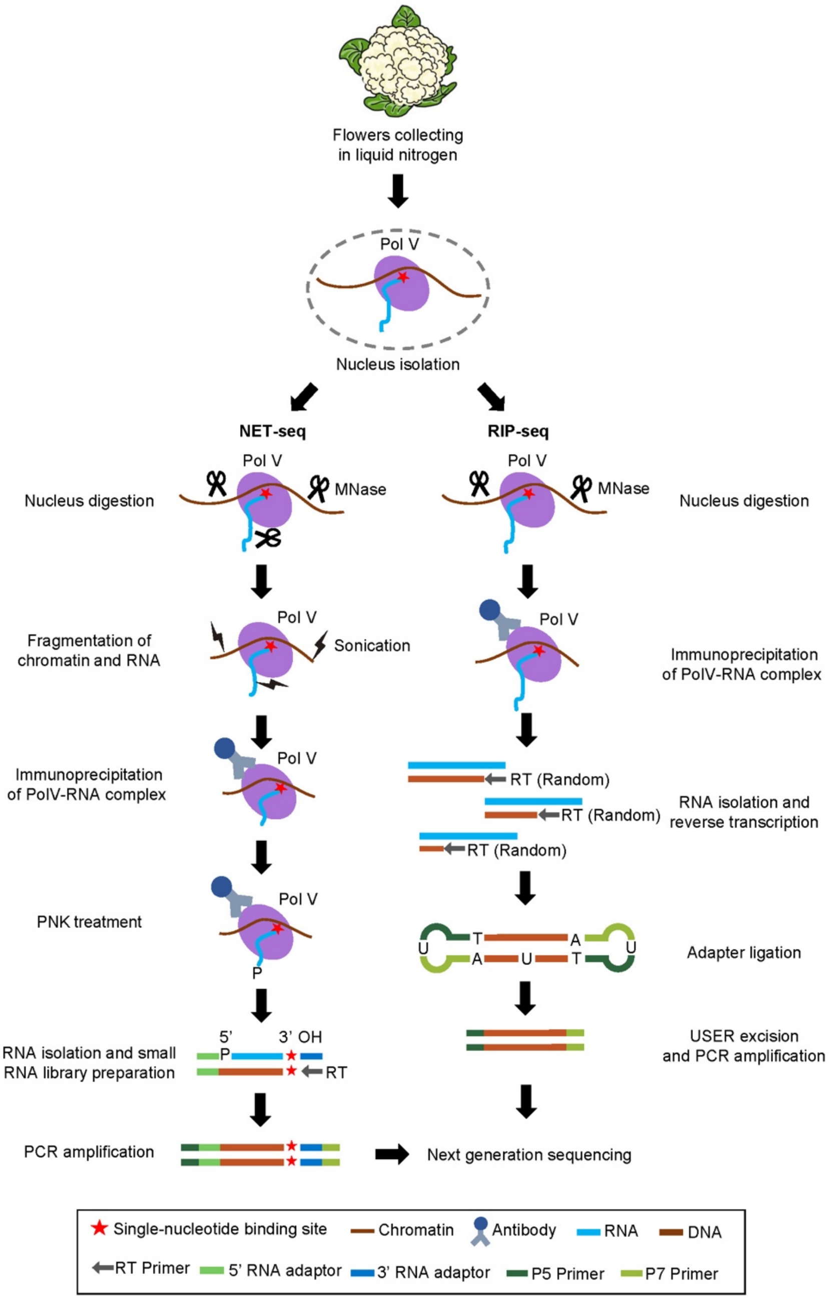

Appendix Figure S1. A diagram of the experimental procedure of Pol V NET-seq and RIP-seq.

In brief, nuclei were isolated from flower tissue and used for NET-seq and RIP-seq. For NET-seq, the RNA was fragmented by MNase treatment and sonication before being subjected to immunoprecipitation using a Pol V antibody. The RNA was adapter-ligated at both ends, reverse-transcribed, and amplified to generate the sequencing library following a small RNA-seq library generation protocol. For RIP-seq, the immunoprecipitation was performed without prior RNA fragmentation by sonication, and RNase inhibitor was added to keep the integrity of the long RNAs. The immunoprecipitated RNA was reverse-transcribed using random primers. The strand-specific sequencing library was then constructed using the dUTP method.

A

	Samples	Raw reads	Clean reads	Mapped reads	Uniquely mapped reads	Remove duplication	Remove rRNA	Remove ChrCM	Remove tRNA	Mean insert length (bp)
Pol V-NET-seq	Rep1	127,470,964	125,001,582	111,376,109	21,908,291	3,735,621	3,689,179	3,642,054	3,640,420	31
	Rep2	135,203,067	133,040,593	121,387,975	24,300,053	4,003,850	3,955,511	3,903,061	3,901,465	31
Pol V-RIP-seq	Rep1	27,816,600	27,633,250	15,420,958	7,369,568	-	7,195,659	7,163,394	7,163,376	240
	Rep2	35,269,266	35,038,541	19,731,972	9,139,382	-	8,922,588	8,882,543	8,882,527	204
	Rep3	24,966,900	24,804,630	14,086,837	6,389,468	-	6,246,238	6,218,974	6,218,932	204

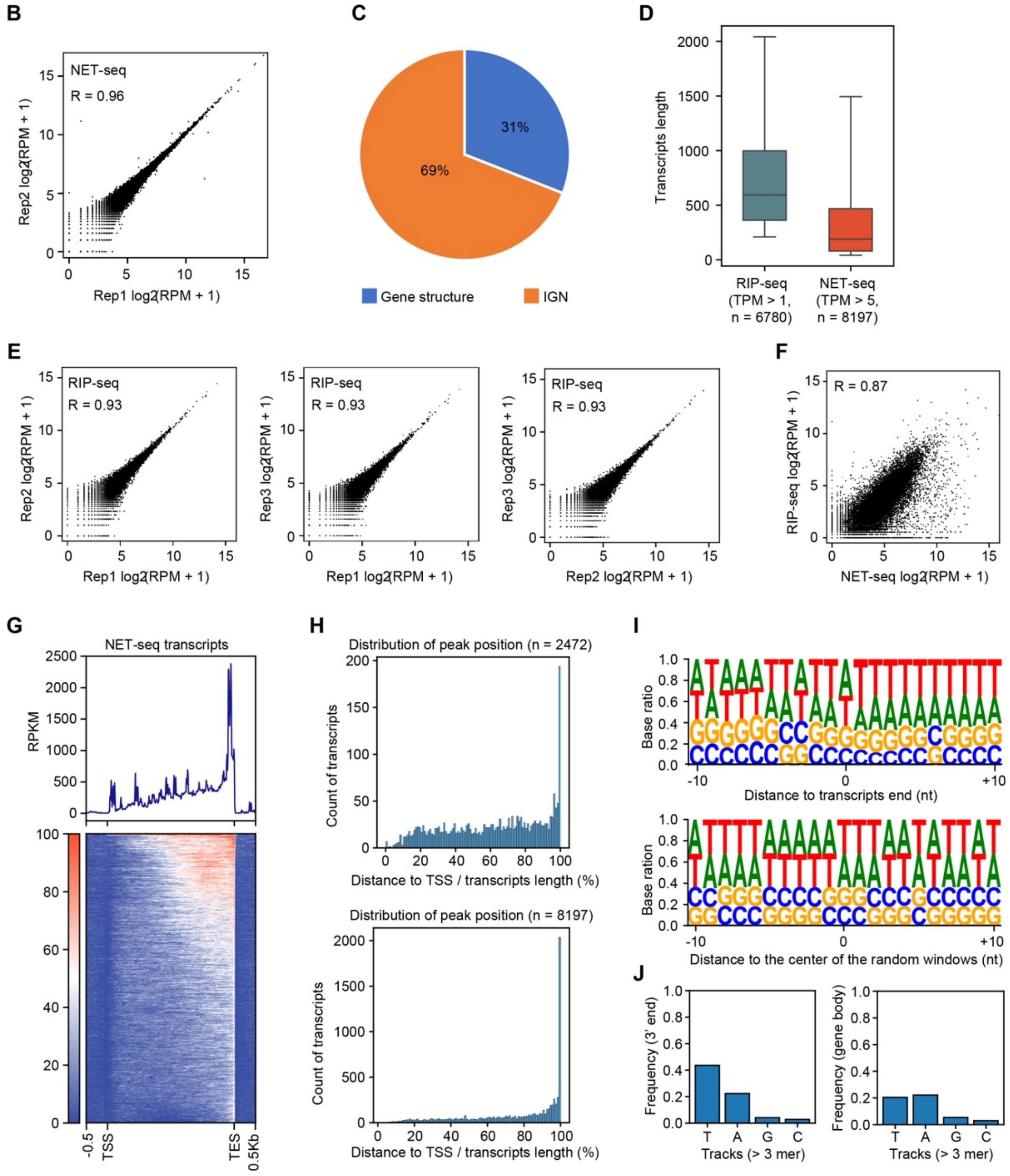

Appendix Figure S2. Statistics of the Pol V NET-seq and RIP-seq data.
(A) Summary of cauliflower Pol V NET-seq and RIP-seq data generated in this study. **(B)** Reproducibility of NET-seq data across the whole genome as calculated by using non-overlapped 10-kb windows for two independent biological replicates. Spearman correlation coefficients are indicated. **(C)** Pie chart showing the ratio of NET-seq reads mapped to gene structure and intergenic region (IGN). **(D)** The distribution of

transcripts length of RIP-seq and NET-seq. Boxplots show the median, with the bottom and top edges of the box representing the 25th and 75th percentiles, respectively. **(E)** Reproducibility of RIP-seq data across the whole genome as calculated by using non-overlapped 10-kb windows for three independent biological replicates. Spearman correlation coefficients are indicated. **(F)** Scatter plot demonstrates the correlation between RIP-seq and NET-seq data across the whole genome as calculated by using non-overlapped 10-kb windows. Replicates are merged to calculate RPM (reads per million). Spearman correlation coefficients are indicated. **(G)** Metagene plot showing the Pol V positioning along all 8,197 NET-seq data assembled transcripts. **(H)** The distribution of the positions with the highest Pol V NET-seq signal along each transcript. Results for 2472 transcripts and all 8197 transcripts were shown in the top and the bottom panels, respectively. **(I)** Sequence logos at the top showing the nucleotide ratio of the 21-nt region around the NET-seq transcript end sites. The x-axis refers to the distance to transcripts end sites; -10 and +10 mean the positions of 10-nt upstream or downstream of transcripts end sites, respectively. The heights of letters reflect the nucleotide ratio at each position calculated from the top 1000 NET-seq transcripts with the highest TPM (transcripts per million). The sequence logos at the bottom showing the nucleotide ratio of randomly selected 21-nt windows within the NET-seq transcript bodies. **(J)** Frequencies of motifs containing at least four consecutive T/A/C/G residues at Pol V transcript 3′ ends (±20 nt, non-template strand, top panel) or within transcript bodies (bottom panel).

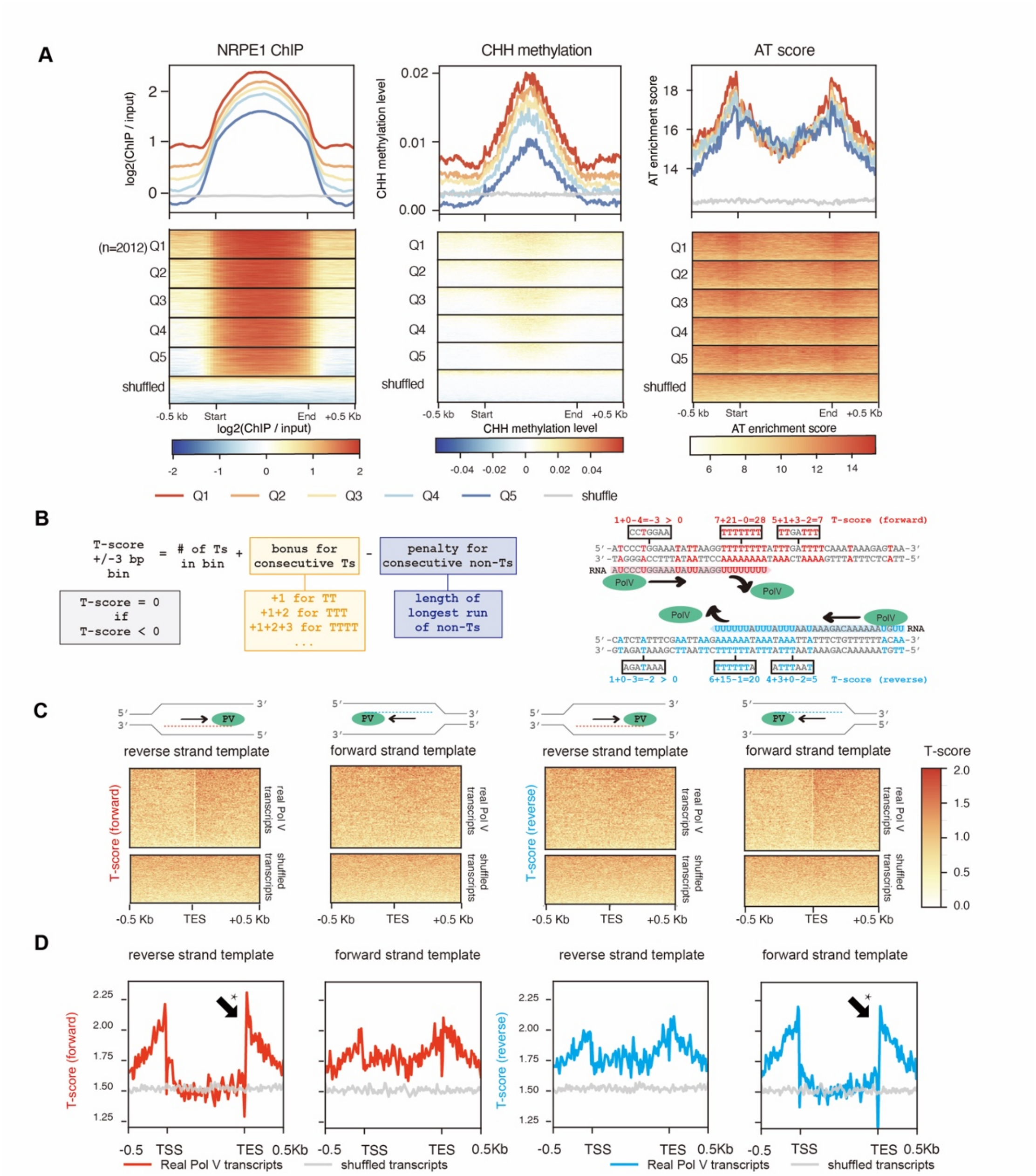

Appendix Figure S3. Arabidopsis Pol V transciption boundaries enrich T-rich sequence.

(A) Metaplot and heatmap representing NRPE1 ChIP-seq signal, AT enrichment signal and CHH methylation signal over NRPE1 ChIP peaks (n=10063) and shuffled regions. The NRPE1 ChIP peaks were evenly divided to five quantiles based on the rank of the NRPE1 ChIP-seq signal. **(B)** Diagram showing the way T-score was calculated on forward and reverse strands. **(C)** Heatmaps over Arabidopsis Pol V transcript end sites (TES) of T-score of either forward (left) or reverse (right) strand. Pol V transcripts transcribed using the forward and reverse strands as template were plotted separately. **(D)** Metaplots over Arabidopsis Pol V transcript from published RIP-seq data, of T-score of either forward (left) or reverse (right) strand. Note that all metaplots are oriented relative to transcription direction, so that the left part of metaplot corresponds to sequences before termination, and the right part after termination. Pol V transcripts

transcribed using the forward and reverse strands as template were plotted separately. *p-value < 2.2e-16, Welch Two-Sample t-test (50 bp regions upstream TES compared to 50 bp regions downstream TES).

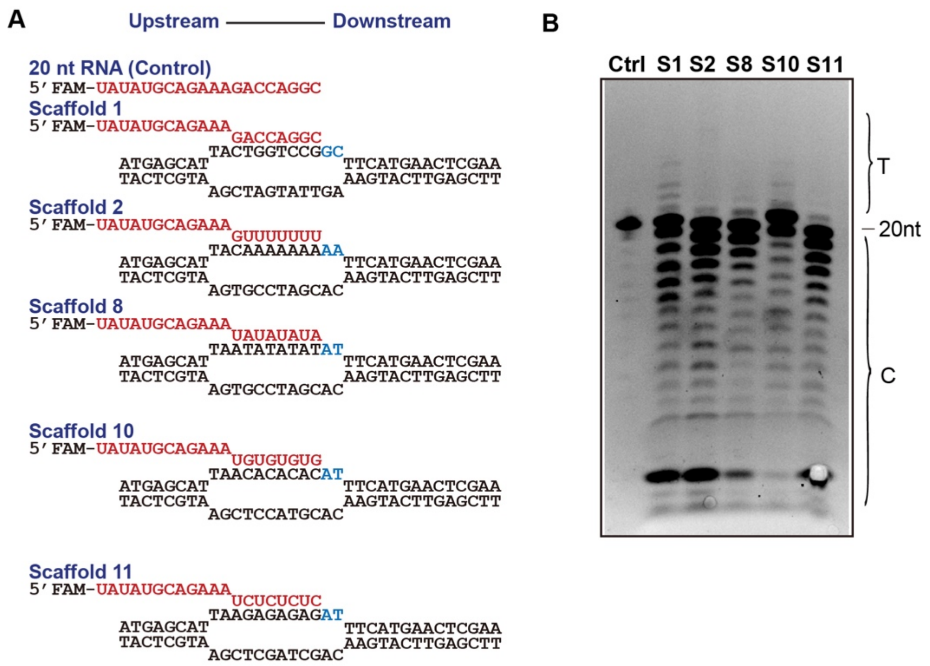

Appendix Figure S4. ***In vitro*** **Pol V transcription assay with designed scaffolds.**
(A) The sequence of the designed scaffold for the *in vitro* assays. **(B)** Transcription assay shows that GA, TA, or CA repeat-containing DNA_T can arrest Pol V elongation or fail to initiate transcription elongation, in a similar way as poly(A)-containing DNA_T. All *in vitro* assays were performed with at least 3 biological repeats, with similar results.

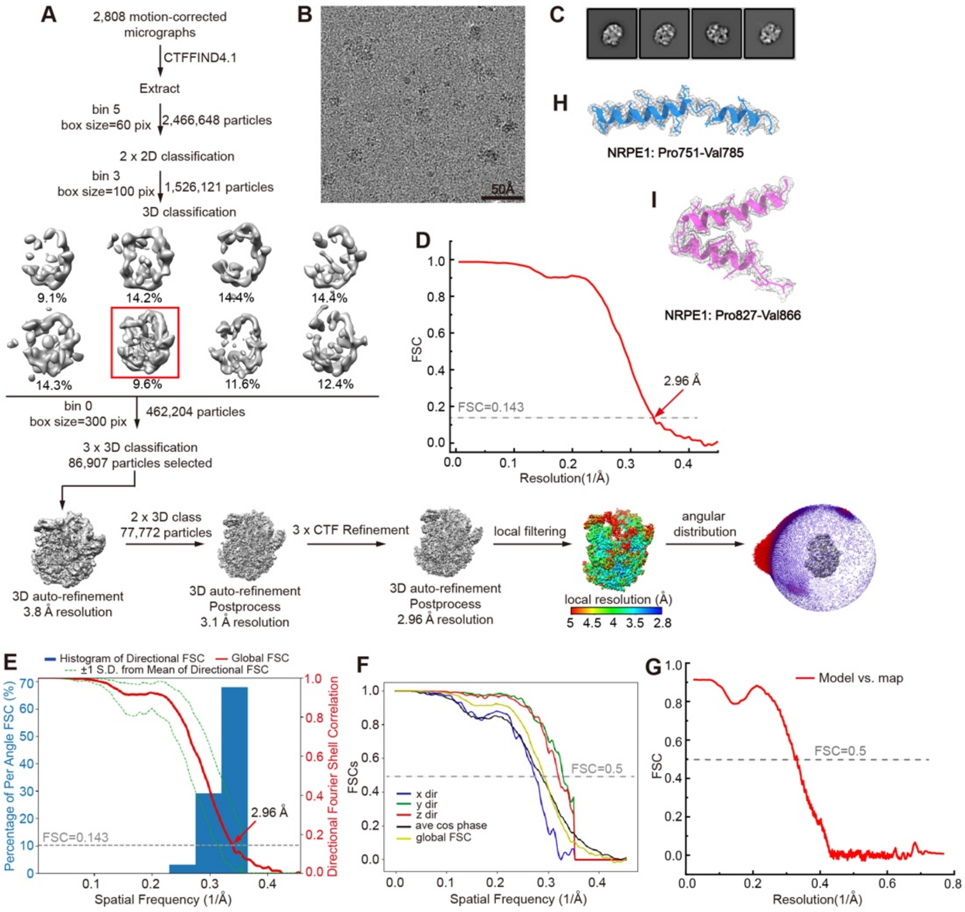

Appendix Figure S5. Cryo-EM structure analysis of the 0U complex.
(A) The flowchart for the data processing, including the local resolution map and angular distribution of particles of the final 3D reconstruction. **(B)** A representative image of the cryo-EM micrograph. **(C)** 2D class averages. **(D)** The FSC curves of the final 3D reconstruction. **(E-F)** The global (E) and directional (F) FSC of the 3D auto-refinement by the 3DFSC Processing Server. **(G)** The FSC calculated between the refined structure and the full map used for refinement. **(H-I)** Electron density maps showed the fitting of representative regions. The map is shown at the same contour level as in Fig. 4C.

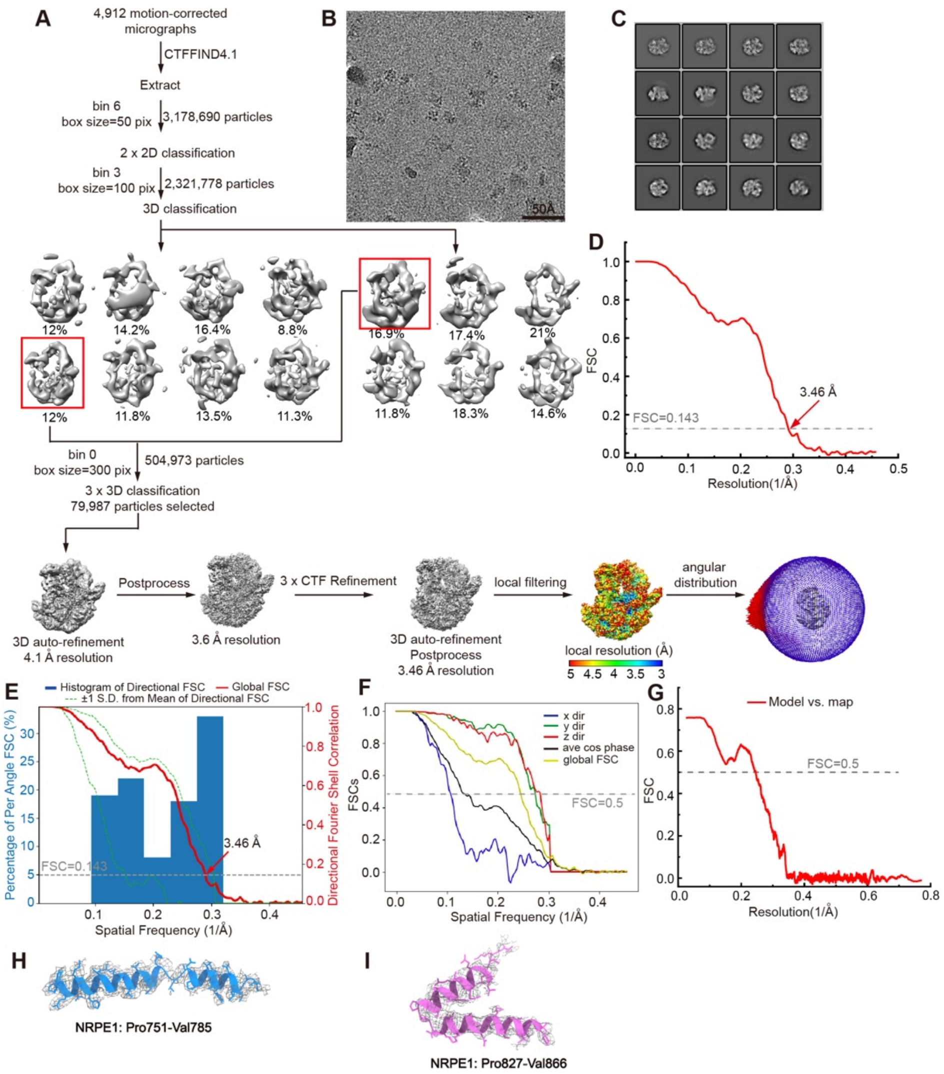

Appendix Figure S6. Cryo-EM structure analysis of the 1U complex.

(A) The flowchart for the data processing, including the local resolution map and angular distribution of particles of the final 3D reconstruction. **(B)** A representative image of the cryo-EM micrograph. **(C)** 2D class averages. **(D)** The FSC curves of the final 3D reconstruction. **(E-F)** The global (E) and directional (F) FSC of the 3D auto-refinement by the 3DFSC Processing Server. **(G)** The FSC calculated between the refined structure and the full map used for refinement. **(H-I)** Electron density maps showed the fitting of representative regions. The map is shown at the same contour level as in Fig. 4D.

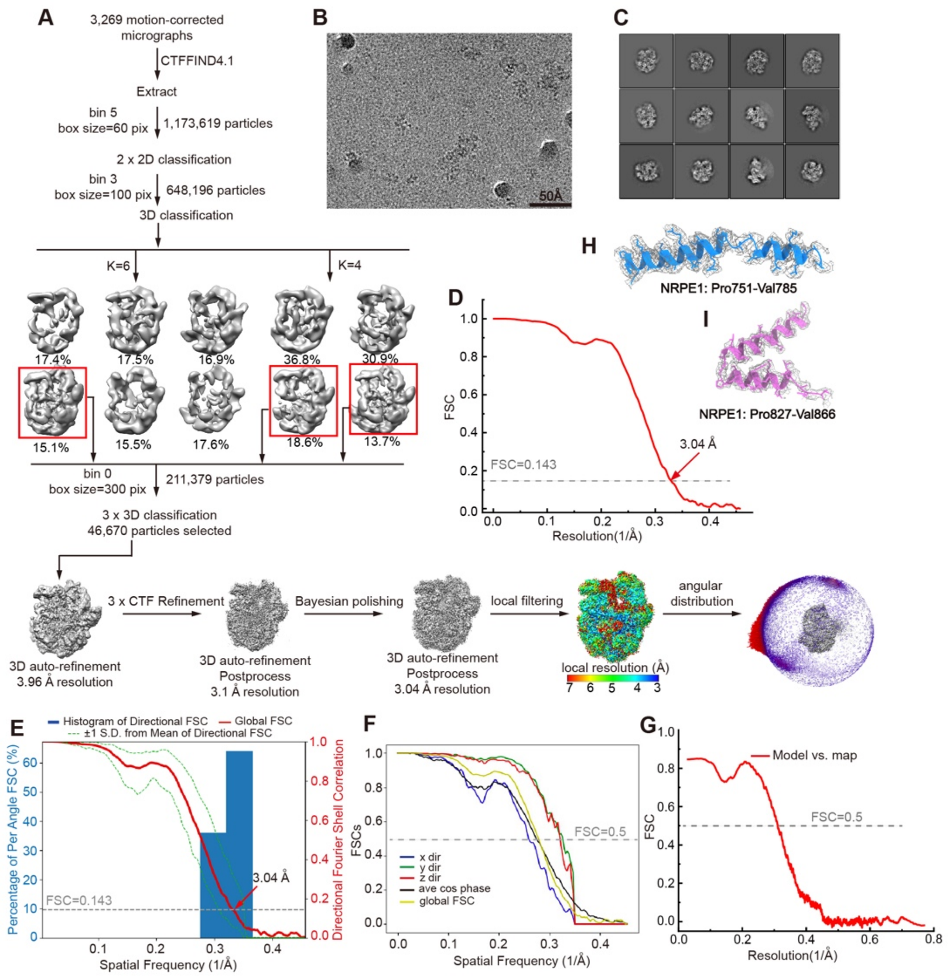

Appendix Figure S7. Cryo-EM structure analysis of the 2U complex.

(A) The flowchart for the data processing, including the local resolution map and angular distribution of particles of the final 3D reconstruction. **(B)** A representative image of the cryo-EM micrograph. **(C)** 2D class averages. **(D)** The FSC curves of the final 3D reconstruction. **(E-F)** The global (E) and directional (F) FSC of the 3D auto-refinement by the 3DFSC Processing Server. **(G)** The FSC calculated between the refined structure and the full map used for refinement. **(H-I)** Electron density maps showed the fitting of representative regions. The map is shown at the same contour level as in Fig. 4E.

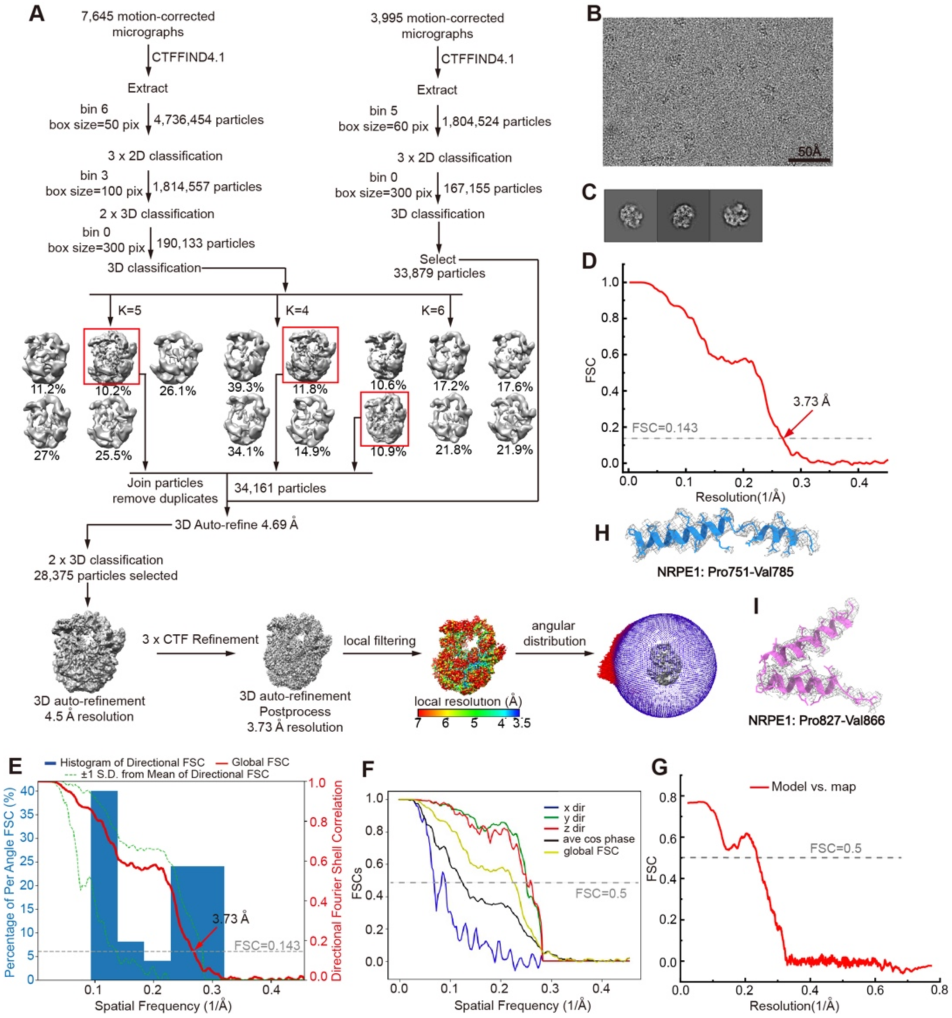

Appendix Figure S8. Cryo-EM structure analysis of the 3U complex.

(A) The flowchart for the data processing, including the local resolution map and angular distribution of particles of the final 3D reconstruction. **(B)** A representative image of the cryo-EM micrograph. **(C)** 2D class averages. **(D)** The FSC curves of the final 3D reconstruction. **(E-F)** The global (E) and directional (F) FSC of the 3D auto-refinement by the 3DFSC Processing Server. **(G)** The FSC calculated between the refined structure and the full map used for refinement. **(H-I)** Electron density maps showed the fitting of representative regions. The map is shown at the same contour level as in Fig. 4F.

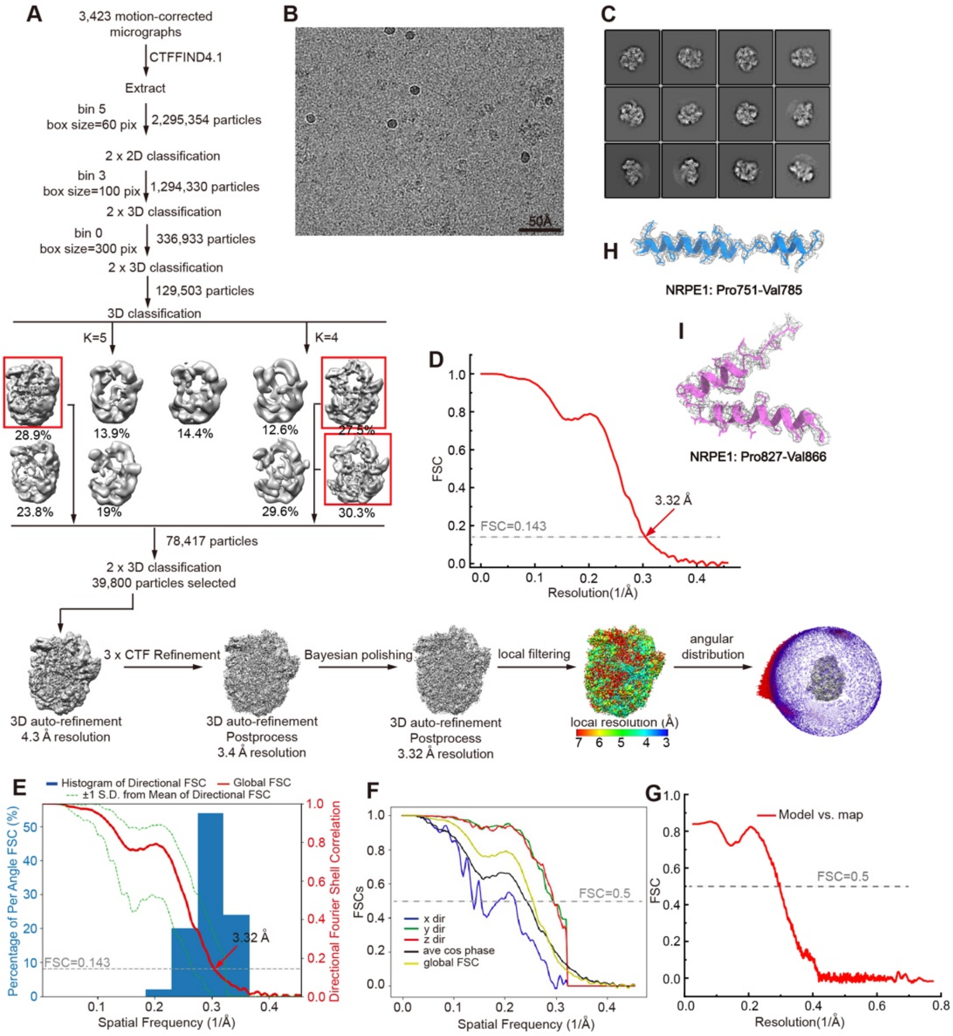

Appendix Figure S9. Cryo-EM structure analysis of the 4U complex.

(A) The flowchart for the data processing, including the local resolution map and angular distribution of particles of the final 3D reconstruction. **(B)** A representative image of the cryo-EM micrograph. **(C)** 2D class averages. **(D)** The FSC curves of the final 3D reconstruction. **(E-F)** The global (E) and directional (F) FSC of the 3D auto-refinement by the 3DFSC Processing Server. **(G)** The FSC calculated between the refined structure and the full map used for refinement. **(H-I)** Electron density maps showed the fitting of representative regions. The map is shown at the same contour level as in Fig. 4G.

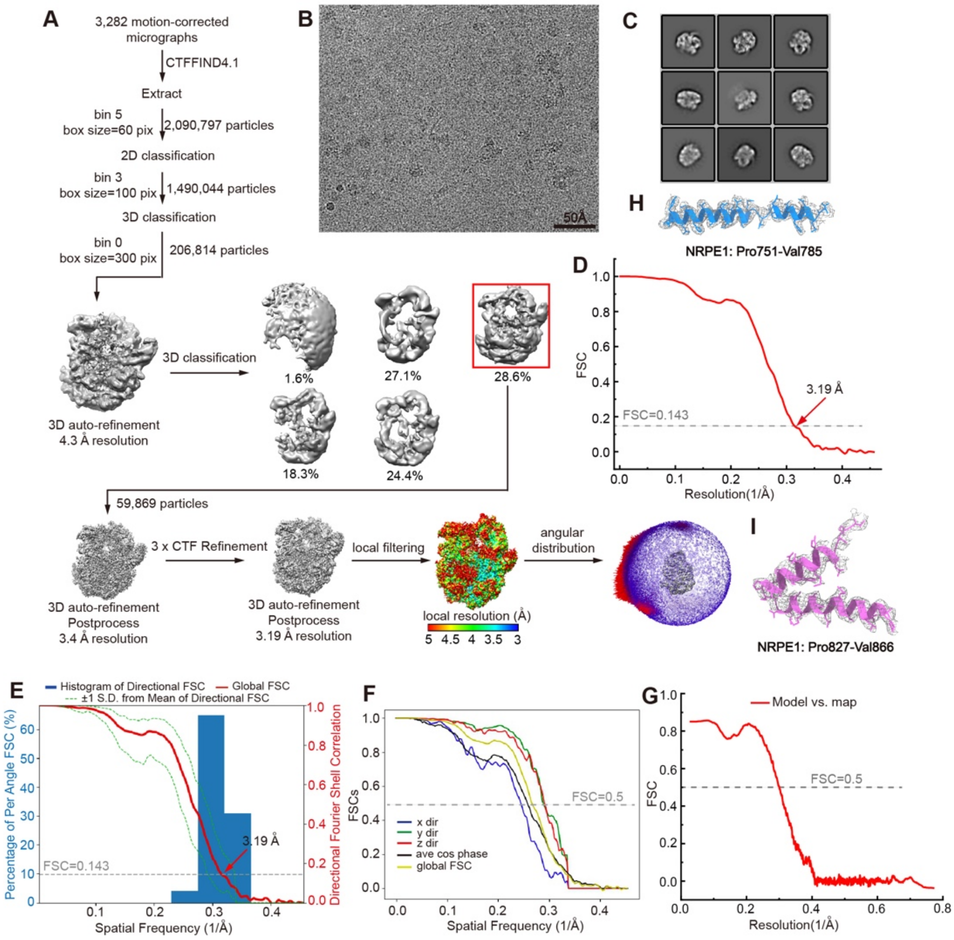

Appendix Figure S10. Cryo-EM structure analysis of the 5U complex.

(A) The flowchart for the data processing, including the local resolution map and angular distribution of particles of the final 3D reconstruction. **(B)** A representative image of the cryo-EM micrograph. **(C)** 2D class averages. **(D)** The FSC curves of the final 3D reconstruction. **(E-F)** The global (E) and directional (F) FSC of the 3D auto-refinement by the 3DFSC Processing Server. **(G)** The FSC calculated between the refined structure and the full map used for refinement. **(H-I)** Electron density maps showed the fitting of representative regions. The map is shown at the same contour level as in Fig. 4H.

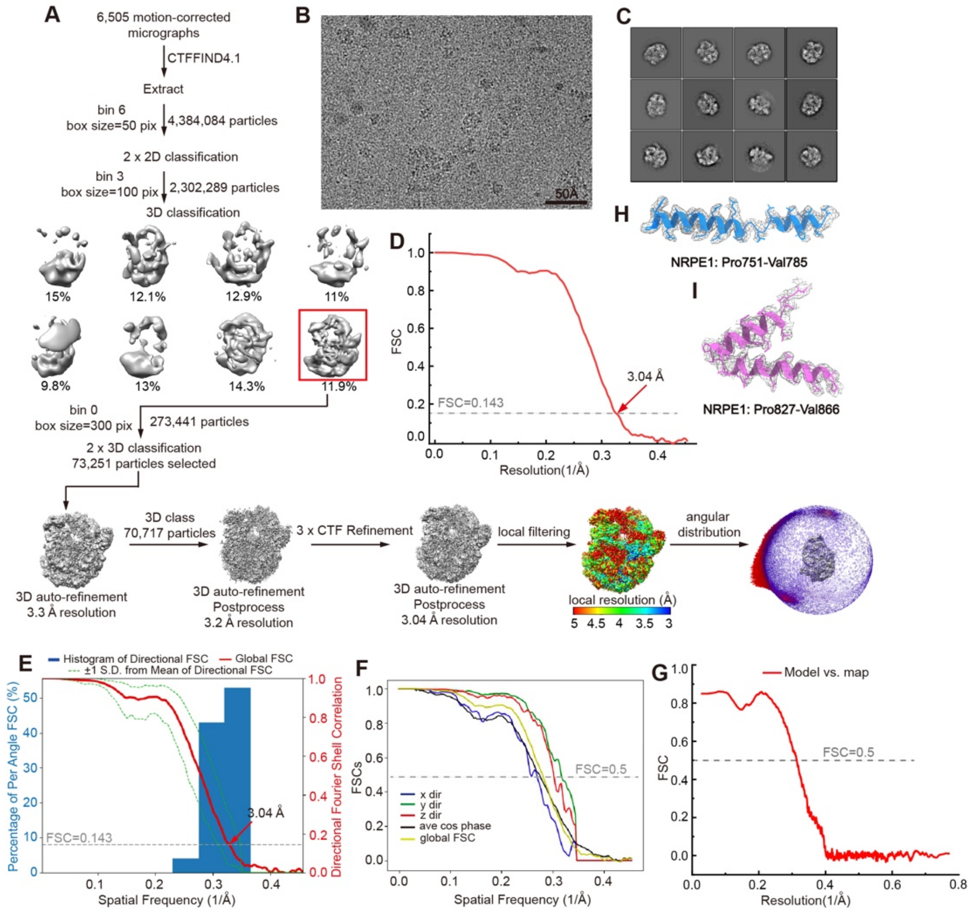

Appendix Figure S11. Cryo-EM structure analysis of the 6U complex.

(A) The flowchart for the data processing, including the local resolution map and angular distribution of particles of the final 3D reconstruction. **(B)** A representative image of the cryo-EM micrograph. **(C)** 2D class averages. **(D)** The FSC curves of the final 3D reconstruction. **(E-F)** The global (E) and directional (F) FSC of the 3D auto-refinement by the 3DFSC Processing Server. **(G)** The FSC calculated between the refined structure and the full map used for refinement. **(H-I)** Electron density maps showed the fitting of representative regions. The map is shown at the same contour level as in Fig. 4I.

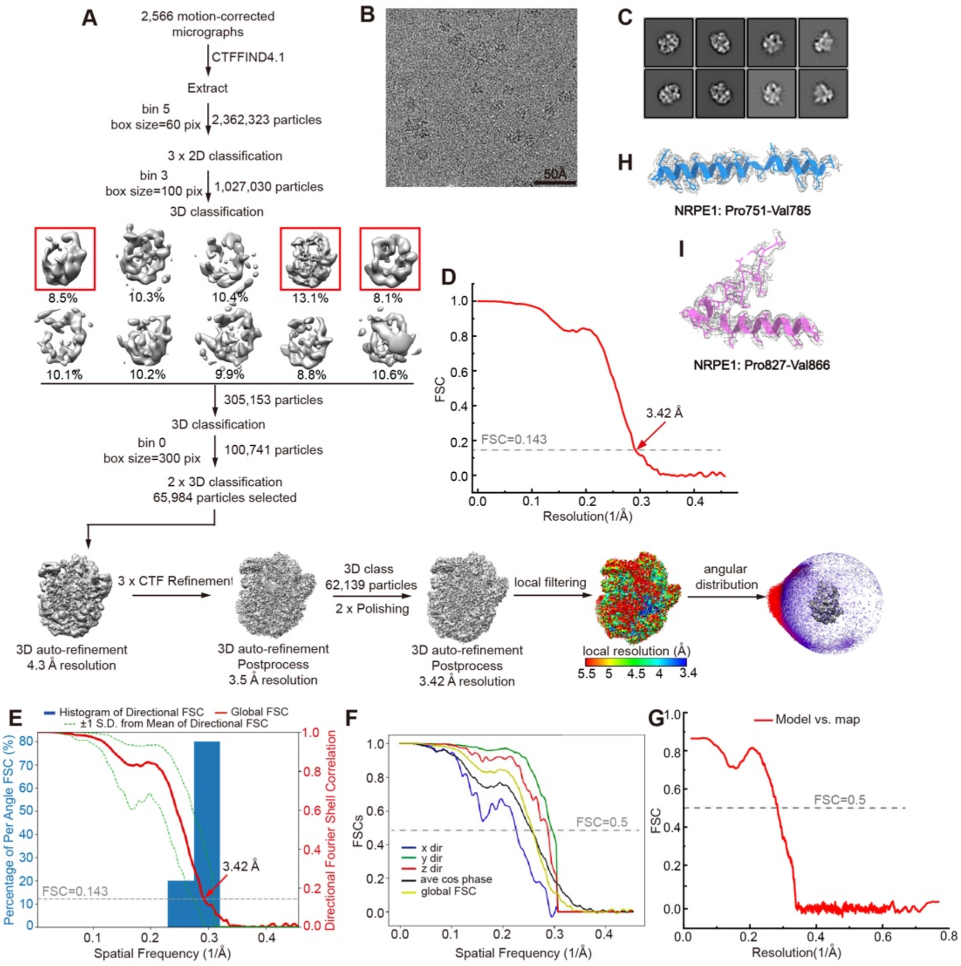

Appendix Figure S12. Cryo-EM structure analysis of the 7U complex.
(A) The flowchart for the data processing, including the local resolution map and angular distribution of particles of the final 3D reconstruction. **(B)** A representative image of the cryo-EM micrograph. **(C)** 2D class averages. **(D)** The FSC curves of the final 3D reconstruction. **(E-F)** The global (E) and directional (F) FSC of the 3D auto-refinement by the 3DFSC Processing Server. **(G)** The FSC calculated between the refined structure and the full map used for refinement. **(H-I)** Electron density maps showed the fitting of representative regions. The map is shown at the same contour level as in Fig. 4J.

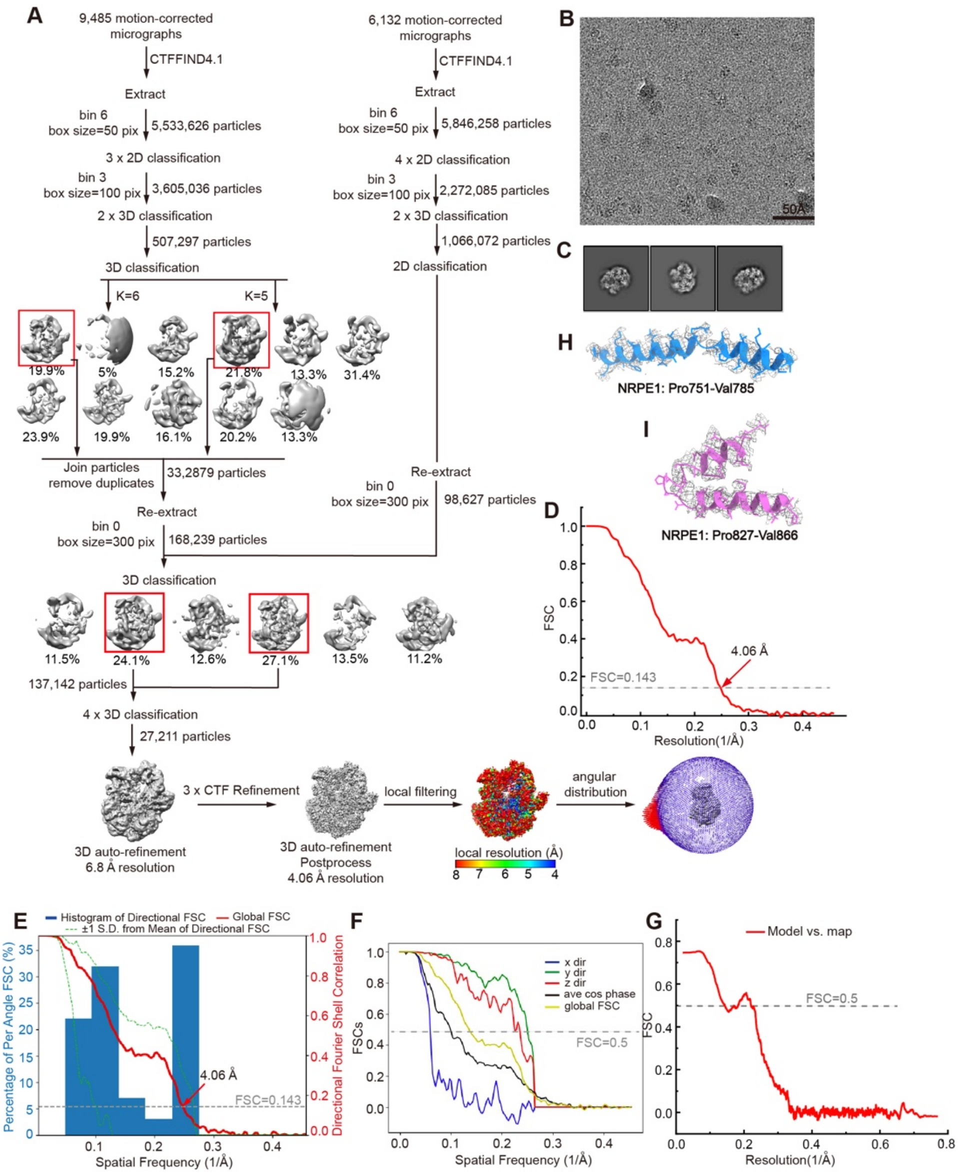

Appendix Figure S13. Cryo-EM structure analysis of the 8U complex.

(A) The flowchart for the data processing, including the local resolution map and angular distribution of particles of the final 3D reconstruction. **(B)** A representative image of the cryo-EM micrograph. **(C)** 2D class averages. **(D)** The FSC curves of the final 3D reconstruction. **(E-F)** The global (E) and directional (F) FSC of the 3D auto-refinement by the 3DFSC Processing Server. **(G)** The FSC calculated between the refined structure and the full map used for refinement. **(H-I)** Electron density maps showed the fitting of representative regions. The map is shown at the same contour level as in Fig. 4K.

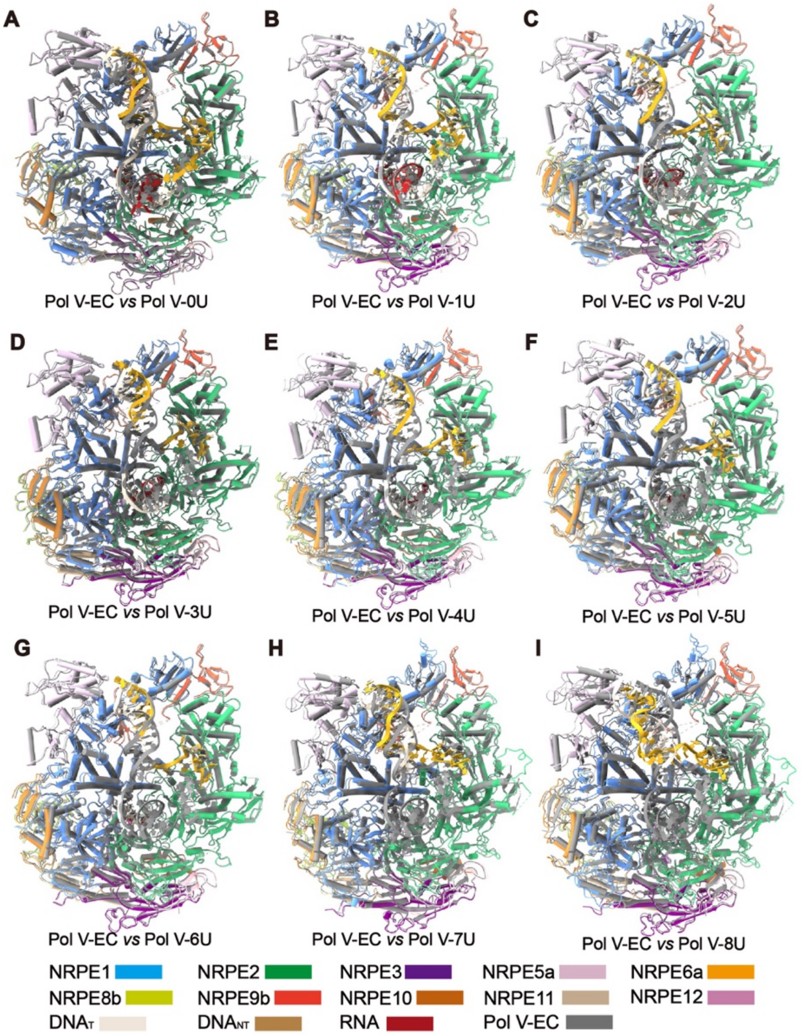

Appendix Figure S14. The structures of 0U to 8U complexes and their comparison with the Pol V elongation conformation.

(A-I) The superimpositions of 0U (A), 1U (B), 2U (C), 3U (D), 4U (E), 5U (F), 6U (G), 7U (H), and 8U (I) complex structures (in color) to the reported Pol V elongation conformation structure (Pol V-EC, in silver, PDB code: 8HIM) revealed almost identical conformations of Pol V during the termination.

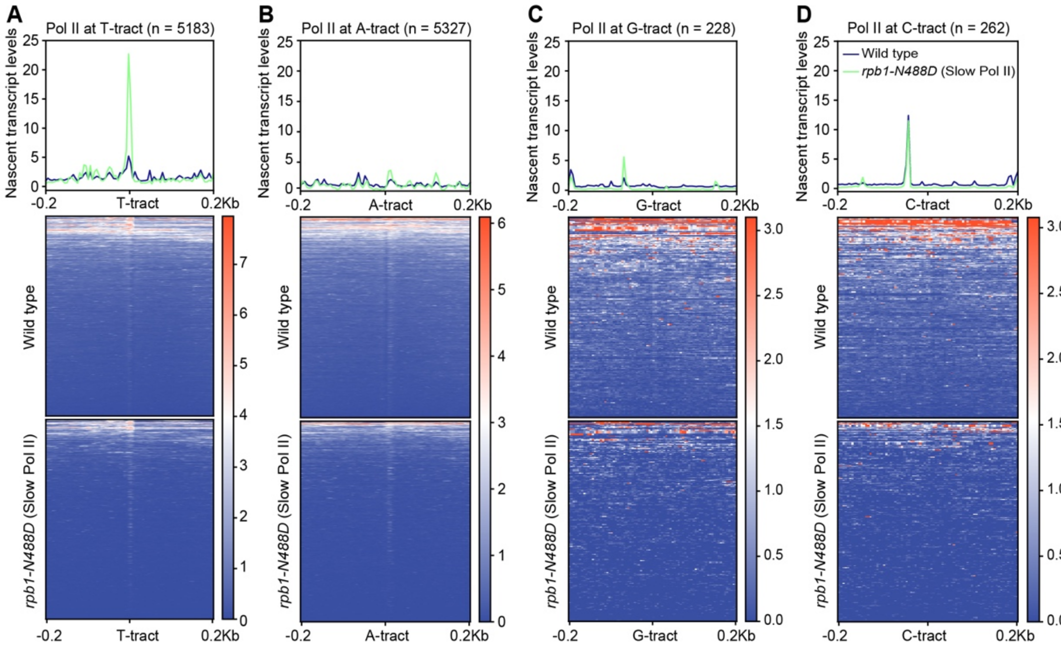

Appendix Figure S15. Slow RNA Pol II of yeast (*S. pombe*) pauses at the T-tract. (A-D) Metaplot showing the signal of wild-type RNA Pol II (blue) and slow Pol II (*rpb1-N488D*, green) NET-seq at non-template strand T-tract (>5 mer) (A), A-tract (>5 mer) (B), G-tract (>5 mer) (C) and C-tract (>5 mer) (D) at gene 3'-end. Poly(T/A/G/C) tracts are identified within 1Kb around the putative polyadenylation site, and the position of the maximum number of repeats was marked as the center for the metaplot.

Appendix Table S1. Oligos used in this research

**Name**	**Sequence**	**Use**
**DNA**		
SF-0U-T	AAGCAAAAAAAAAACGGCCTGGTCATTACGAGTA	EM
SF-0U-NT	TACTCGTAAGCTAGTATTGATTTTTTTTTTGCTT	EM
SF-1U-T	AAGCTCAAGTACTTAAAGCGTCGCATTACGAGTA	EM
SF-1U-NT	TACTCGTAAGTGCCTAGCACAAGTACTTGAGCTT	EM
SF-2U-T	AAGCTCAAGTACTTAAAACGTCGCATTACGAGTA	EM
SF-2U-NT	TACTCGTAAGTGCCTAGCACAAGTACTTGAGCTT	EM
SF-3U-T	AAGCTCAAGTACTTAAAAAGTCGCATTACGAGTA	EM
SF-3U-NT	TACTCGTAAGTGCCTAGCACAAGTACTTGAGCTT	EM
SF-4U-T	AAGCTCAAGTACTTAAAAAATCGCATTACGAGTA	EM
SF-4U-NT	TACTCGTAAGTGCCTAGCACAAGTACTTGAGCTT	EM
SF-5U-T	AAGCTCAAGTACTTAAAAAAACGCATTACGAGTA	EM
SF-5U-NT	TACTCGTAAGTGCCTAGCACAAGTACTTGAGCTT	EM
SF-6U-T	AAGCTCAAGTACTTAAAAAAAAGCATTACGAGTA	EM
SF-6U-NT	TACTCGTAAGTGCCTAGCACAAGTACTTGAGCTT	EM
SF-7U-T	AAGCTCAAGTACTTAAAAAAAAACATTACGAGTA	EM
SF-7U-NT	TACTCGTAAGTGCCTAGCACAAGTACTTGAGCTT	EM
SF-8U-T	AAGCTCAAGTACTTAAAAAAAAAAATTACGAGTA	EM
SF-8U-NT	TACTCGTAAGAGCCTAGCACAAGTACTTGAGCTT	EM
SF-1-T	AAGCTCAAGTACTTCGGCCTGGTCATTACGAGTA	Assay
SF-1-NT	TACTCGTAAGCTAGTATTGAAAGTACTTGAGCTT	Assay
SF-2-T	AAGCTCAAGTACTTAAAAAAAAACATTACGAGTA	Assay
SF-2-NT	TACTCGTAAGTGCCTAGCACAAGTACTTGAGCTT	Assay
SF-3-T	AAGCTCAAGTACTTCGTCCTGGTCATTACGAGTA	Assay
SF-3-NT	TACTCGTAAGTTTTTTTTTTAAGTACTTGAGCTT	Assay
SF-4-T	AAGCTCAAGTACTTCGACCAGGACATTACGAGTA	Assay
SF-4-NT	TACTCGTAAGAAAAAAAAAAAAGTACTTGAGCTT	Assay
SF-5-T	AAGCAAAAAAAAAACGGCCTGGTCATTACGAGTA	Assay
SF-5-NT	TACTCGTAAGCTAGTATTGATTTTTTTTTTGCTT	Assay
SF-6-T	AAGCTTTTTTTTTTCGGCCTGGTCATTACGAGTA	Assay
SF-6-NT	TACTCGTAAGCTAGTATTGAAAAAAAAAAAGCTT	Assay
SF-7-T	AAGCTCAAGTACTTTTTTTTTTTCATTACGAGTA	Assay
SF-7-NT	TACTCGTAAGTGCCTAGCACAAGTACTTGAGCTT	Assay
SF-8-T	AAGCTCAAGTACTTTATATATATAATTACGAGTA	Assay
SF-8-NT	TACTCGTAAGTGCCTAGCACAAGTACTTGAGCTT	Assay
SF-9-T	AAGCTCAAGTACTTTATTTTAAAAATTACGAGTA	Assay
SF-9-NT	TACTCGTAAGTGCCTAGCACAAGTACTTGAGCTT	Assay
**RNA**		
RNA-0U	UAUAUGCAGAAAGACCAGGC	EM
RNA-1U	UAUAUGCAGAAAGCGACGCU	EM
RNA-2U	UAUAUGCAGAAAGCGACGUU	EM

RNA-3U	UAUAUGCAGAAAGCGACUUU	EM
RNA-4U	UAUAUGCAGAAAGCGAUUUU	EM
RNA-5U	UAUAUGCAGAAAGCGUUUUU	EM
RNA-6U	UAUAUGCAGAAAGCUUUUUU	EM
RNA-7U	UAUAUGCAGAAAGUUUUUUU	EM
RNA-8U	UAUAUGCAGAAAUUUUUUUU	EM
RNA-20nt	FAM-UAUAUGCAUAAAGACCAGGC	Assay
RNA-SF-1	FAM-UAUAUGCAGAAAGACCAGGC	Assay
RNA-SF-2	FAM-UAUAUGCAGAAAGUUUUUUU	Assay
RNA-SF-3	FAM-UAUAUGCAGAAAGACCAGGA	Assay
RNA-SF-4	FAM-UAUAUGCAGAAAGUCCUGGU	Assay
RNA-SF-5	FAM-UAUAUGCAGAAAGACCAGGC	Assay
RNA-SF-6	FAM-UAUAUGCAGAAAGACCAGGC	Assay
RNA-SF-7	FAM-UAUAUGCAGAAAGAAAAAAA	Assay
RNA-SF-8	FAM-UAUAUGCAGAAAUAUAUAUA	Assay
RNA-SF-9	FAM-UAUAUGCAGAAAUUUUAAAA	Assay

Appendix Table S2. Cryo-EM data collection, refinement, and validation statistics.

	0U complex	1U complex	2U complex	3U complex	4U complex	5U complex	6U complex	7U complex	8U complex
EMDB code	EMD-61961	EMD-61962	EMD-61963	EMD-61964	EMD-61965	EMD-61966	EMD-61967	EMD-61968	EMD-61969
PDB code	9K11	9K12	9K13	9K14	9K15	9K16	9K17	9K18	9K19
**Data collection and processing**									
Microscopy	Titan Krios (SUSTech #2)	Titan Krios (PKU-IAAS)	Titan Krios (PKU-IAAS)	Titan Krios (PKU-IAAS)	Titan Krios (PKU-IAAS)	Titan Krios (PKU-IAAS)	Titan Krios (PKU-IAAS)	Titan Krios (SUSTech #2)	Titan Krios (SUSTech #2)
Voltage (kV)	300	300	300	300	300	300	300	300	300
Camera	Gatan K3	Gatan K3	Gatan K3	Gatan K3	Gatan K3	Gatan K3	Gatan K3	Gatan K3	Gatan K3
Magnification	81,000	81,000	81,000	81,000	81,000	81,000	81,000	81,000	81,000
Pixel size (Å/pixel)	1.095	1.095	1.095	1.095	1.095	1.095	1.095	1.095	1.095
Electron exposure ($e^-/Å^2$)	50	50	50	50	50	50	50	50	50
Exposure rate (e^-/A^2/sec)	20	20	20	20	20	20	20	20	20
Frames per movie (no.)	32	32	32	32	32	32	32	32	32
Defocus range (μm)	-1.0 to -2.5	-1.0 to -2.5	-1.0 to -2.5	-1.0 to -2.5	-1.0 to -2.5	-1.0 to -2.5	-1.0 to -2.5	-1.0 to -2.5	-1.0 to -2.5
Automation software	EPU	EPU	EPU	EPU	EPU	EPU	EPU	EPU	EPU
Energy filter slit width (eV)	20	20	20	20	20	20	20	20	20
Micrographs (no.)	2,808	4,912	3,269	7,645<br>3,995	3,423	3,282	6,505	2,566	9,485<br>6,132
Micrographs used (no.)	2,808	4,912	3,269	11,640	3,423	3,282	6,505	2,566	15,617
Total Extracted particles (no.)	2,466,648	3,178,690	1,173,619	4,736,454<br>1,804,524	2,295,354	2,090,797	4,384,084	2,362,323	5,533,626<br>5,846,258
**For each reconstruction**									
Refined particles (no.)	77,772	79,987	46,670	28,375	39,800	59,869	70,717	62,139	27,211
Final particles (no.)	77,772	79,987	46,670	28,375	39,800	59,869	70,717	62,139	27,211
Data processing software	Relion3.1	Relion3.1	Relion3.1	Relion3.1	Relion3.1	Relion3.1	Relion3.1	Relion3.1	Relion3.1

Point group	*C*1	*C*1	*C*1	*C*1	*C*1	*C*1	*C*1	*C*1	*C*1
Resolution (global, Å)	2.96	3.46	3.04	3.73	3.32	3.19	3.04	3.42	4.06
FSC 0.143 (unmasked/masked)	3.8/2.96	4.13/3.46	4.03/3.04	4.59/3.73	4.17/3.32	3.96/3.19	3.84/3.04	4.21/3.42	7.46/4.06
FSC 0.5 (unmasked/masked)	7.3/3.43	8.22/4.11	8.2/3.61	9.9/4.5	8.4/3.98	7.81/3.79	7.14/3.57	8.06/3.91	11.36/7.46
Local resolution range (Å)	2.8-5.0	3.0-5.0	3.0-7.0	3.5-7.0	3.0-7.0	3.0-5.0	3.0-5.0	3.4-5.5	4.0-8.0
Map sharpening B-factor (Å^2)	-35	-60	-30	-60	-57	-30	-40	-56	-60
Map sharpening methods	Half-maps correlation	Half-maps correlation	Half-maps correlation	Half-maps correlation	Half-maps correlation	Half-maps correlation	Half-maps correlation	Half-maps correlation	Half-maps correlation
**Refinement**									
Refinement package	Phenix	Phenix	Phenix	Phenix	Phenix	Phenix	Phenix	Phenix	Phenix
- real or reciprocal space	Real space	Real space	Real space	Real space	Real space	Real space	Real space	Real space	Real space
- resolution cutoff (Å)	3.0	3.5	3.04	3.8	3.3	3.19	3.04	3.5	4.1
Model-Map scores									
- CC	0.85	0.62	0.77	0.61	0.78	0.81	0.82	0.80	0.58
No. atoms									
Protein / nucleotides	22,609/1,307	22,512/1307	22,575/908	22,431/768	22,612/485	22,564/615	22,568/574	23,005/696	22,734/653
Mg^{2+} / Zn^{2+}	1/6	1/6	1/6	1/6	1/6	1/6	1/6	1/6	1/6
B-factors (Å^2)									
Protein / nucleotides	88.7/124.4	134.4/113.0	81.6/67.4	111.0/92.6	77.0/121.4	105.8/124.1	101.9/105.2	88.4/77.3	121.4/125.2
Mg^{2+} / Zn^{2+}	141.1	189.9	167.0	184.7	161.0	200.3	224.5	117.6	137.7
R.m.s. deviations									
Bond lengths (Å)	0.003	0.003	0.003	0.005	0.004	0.003	0.003	0.004	0.005
Bond angles (°)	0.621	0.684	0.647	0.817	0.599	0.664	0.647	0.677	0.889

**Validation**									
MolProbity score	1.65	1.73	1.67	2.17	1.99	1.59	1.57	2.10	2.24
Clashscore	5.40	7.88	5.39	12.96	6.17	6.08	5.47	6.44	13.83
Poor rotamers (%)	0.80	0.84	1.23	1.40	2.36	0.84	1.12	1.79	0.00
C-beta outlier (%)	0.00	0.00	0.00	0.00	0.00	0.00	0.00	0.00	0.00
CaBLAM outliers	2.93	2.54	2.54	3.19	2.42	2.94	2.61	5.62	6.47
Ramachandran plot									
Favored (%)	94.8	95.7	95.6	93.4	94.6	96.2	96.4	89.8	88.2
Allowed (%)	5.2	4.3	4.4	6.6	5.4	3.8	3.6	10.2	11.7
Outlier (%)	0.0	0.0	0.0	0.0	0.0	0.0	0.0	0.0	0.1
