# Supplementary material for: A spontaneous termination mechanism of RNA polymerase V shapes the DNA methylation landscape in plants
Source: EMBO J. 2026 Apr 2;45(9):3192–205. doi: 10.1038/s44318-026-00763-7 (PMC13144423; doi:10.1038/s44318-026-00763-7)
Supplement: Supplementary file 3 — Figure S4 Source Data [file 44318_2026_763_MOESM3_ESM.zip › Source data Appendix Fig. S4.pptx]

## Slide 1
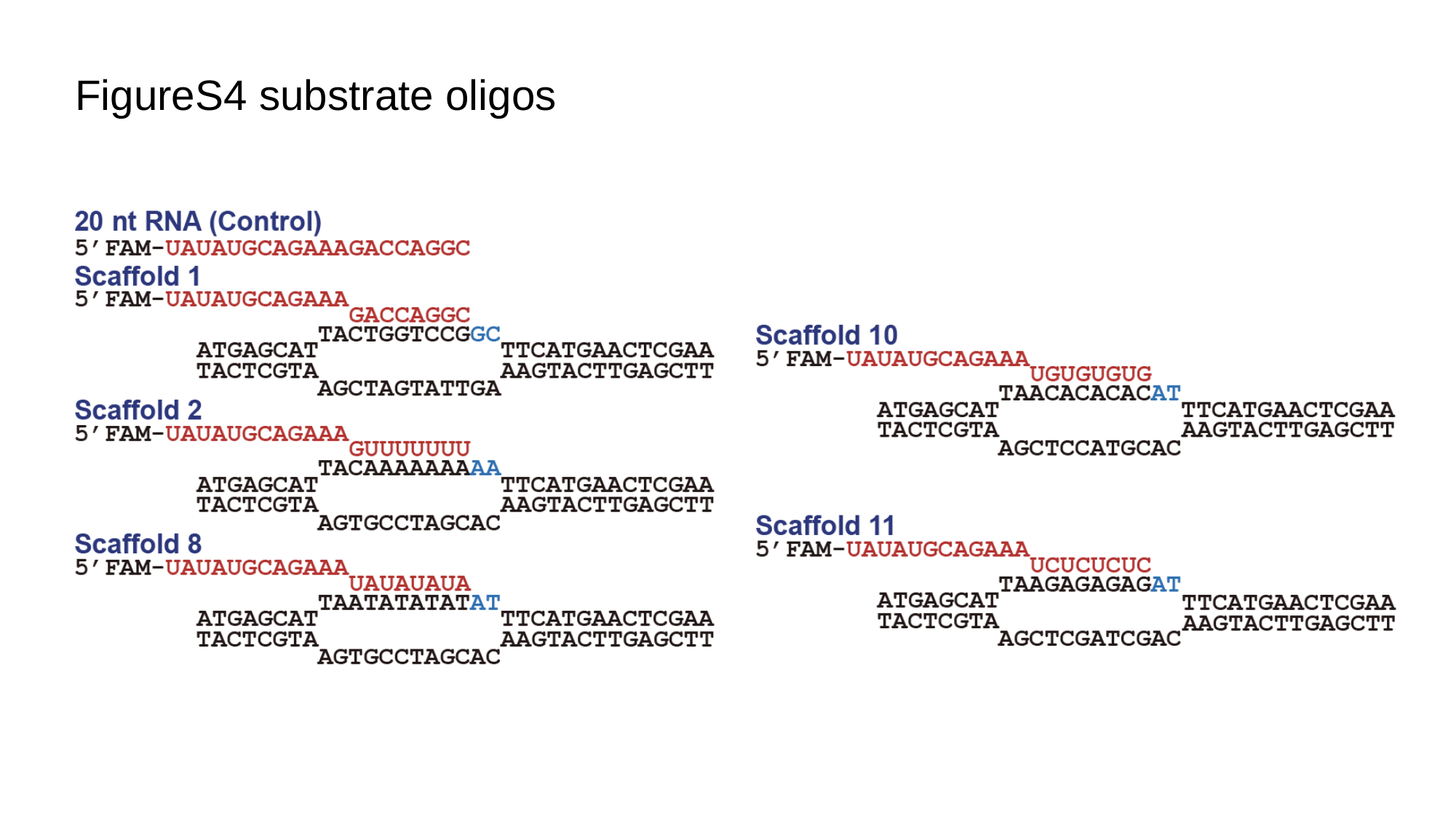

FigureS4 substrate oligos

## Slide 2
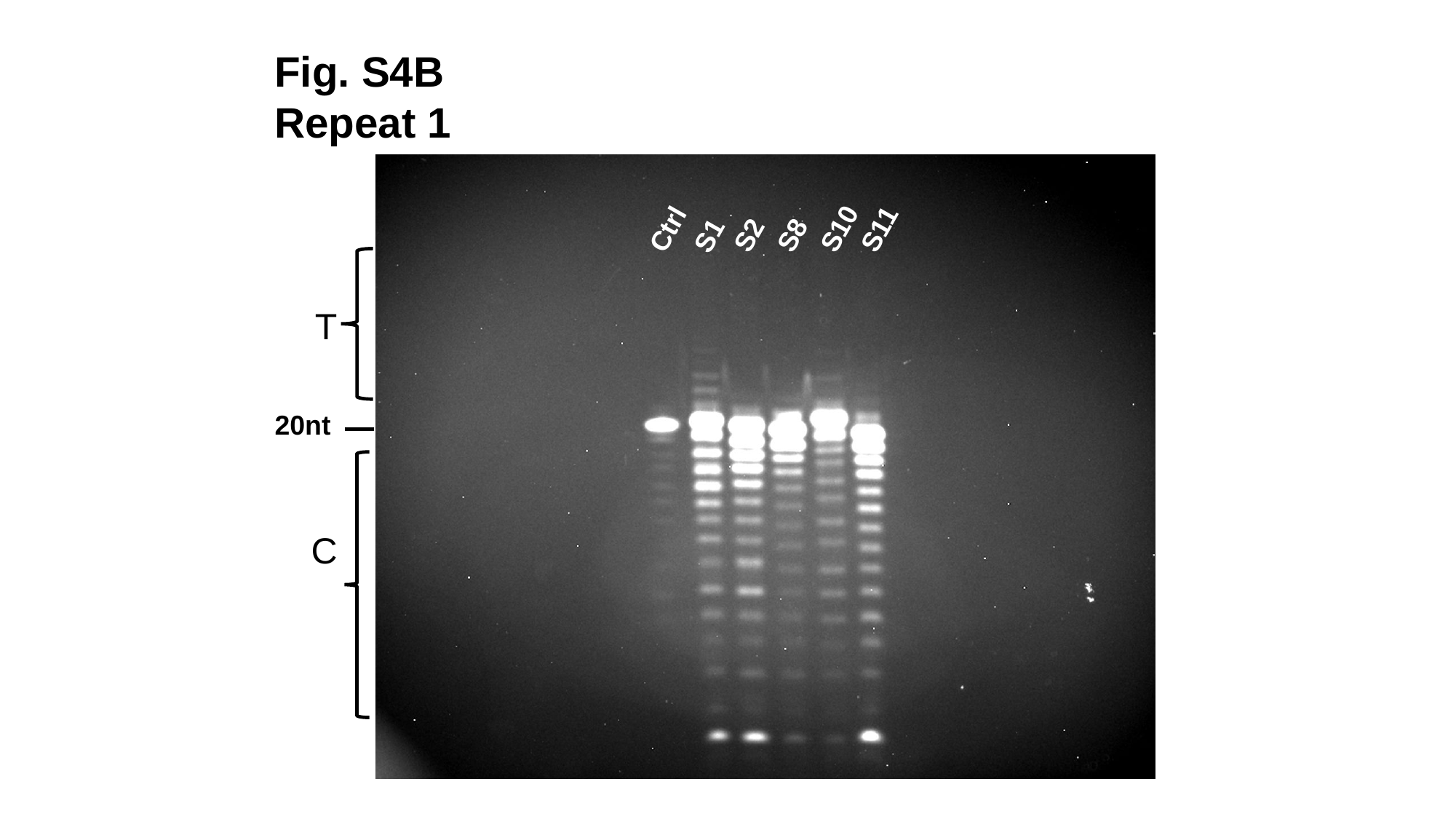

Fig. S4B
Repeat 1
Ctrl
S2
S8
S10
S11
S1
T
20nt
C

## Slide 3
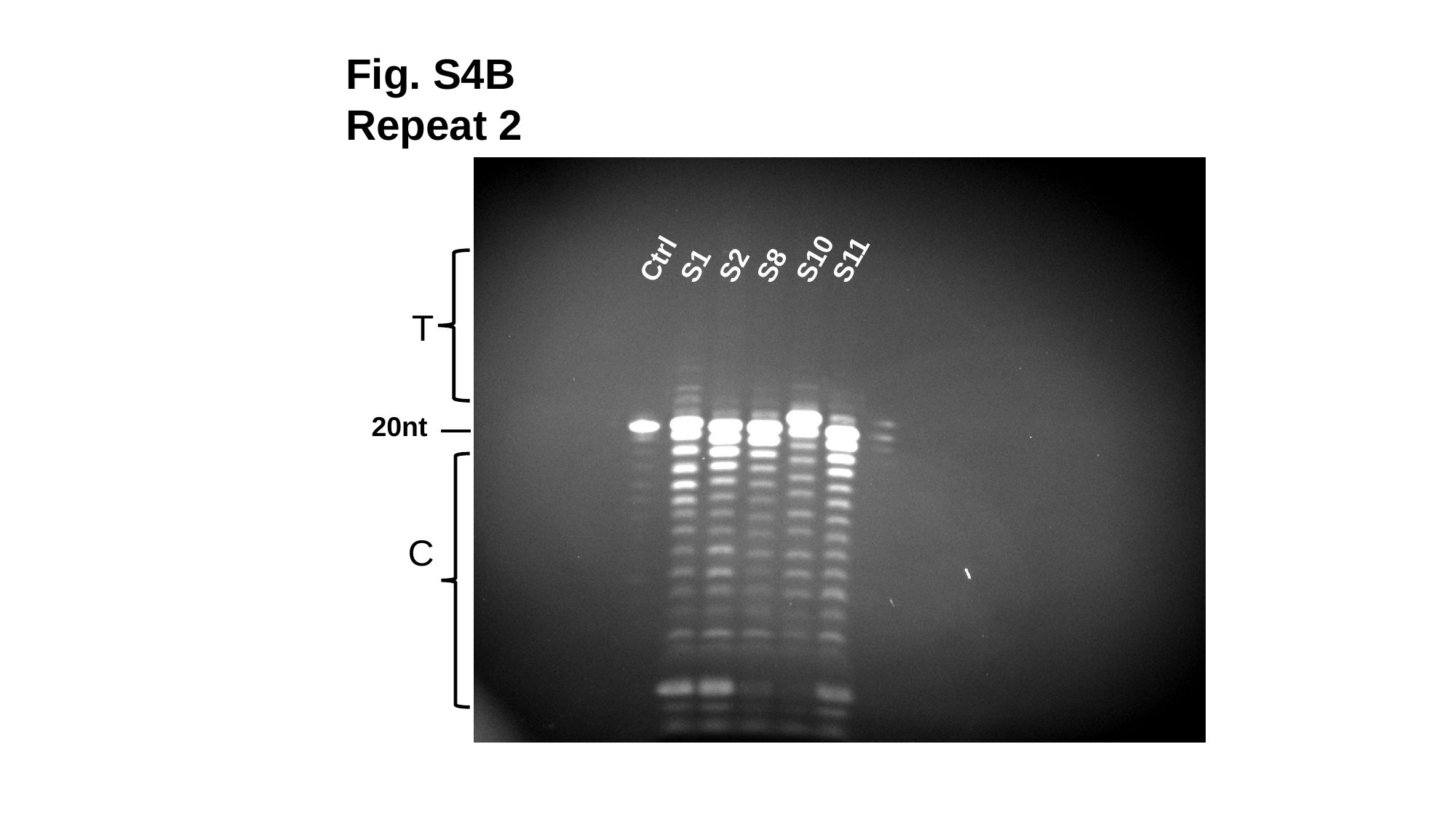

Fig. S4B
Repeat 2
Ctrl
S2
S8
S10
S11
S1
T
20nt
C

## Slide 4
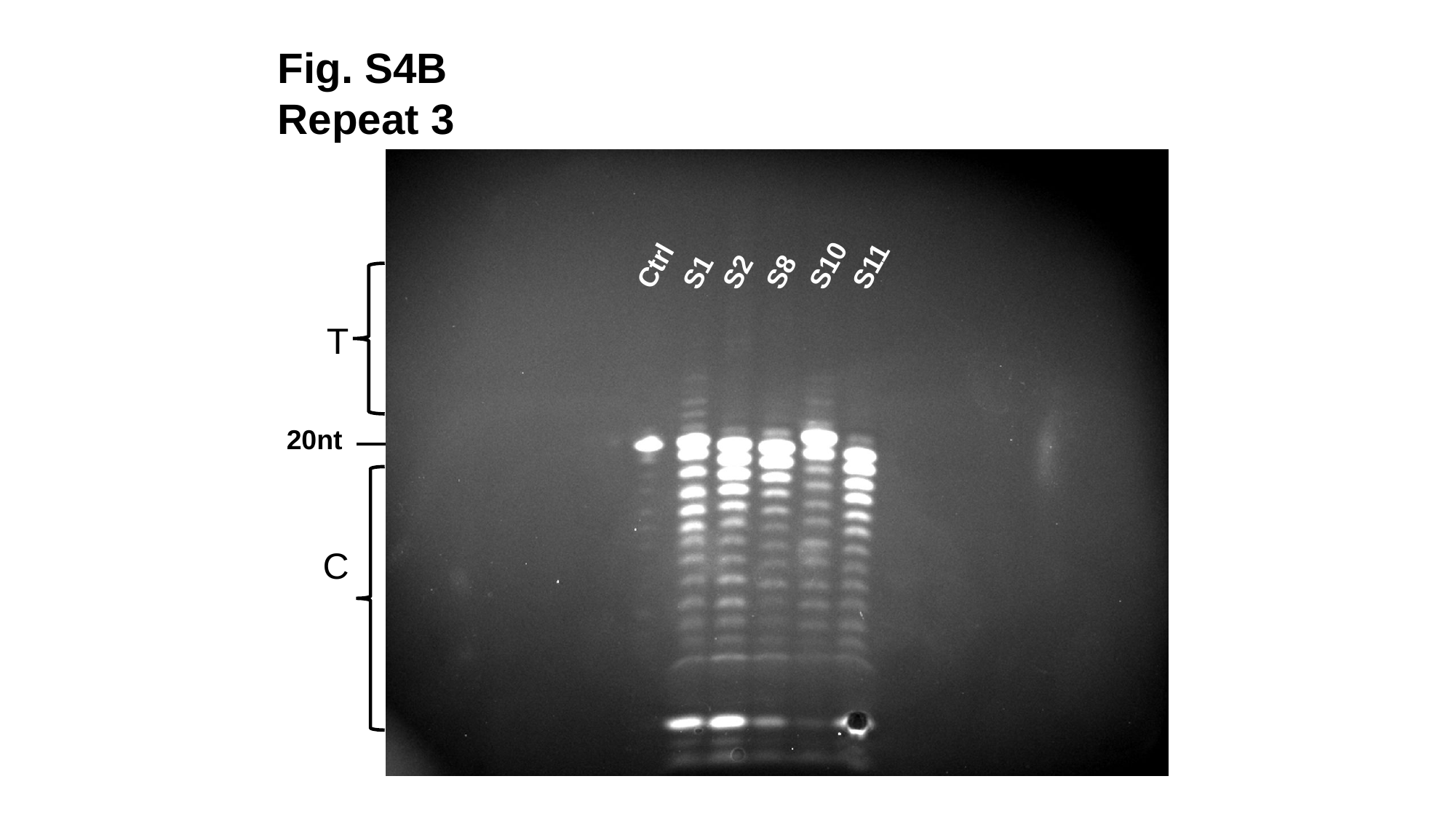

Fig. S4B
Repeat 3
Ctrl
S2
S8
S10
S11
S1
T
20nt
C
