# Supplementary material for: A spontaneous termination mechanism of RNA polymerase V shapes the DNA methylation landscape in plants
Source: EMBO J. 2026 Apr 2;45(9):3192–205. doi: 10.1038/s44318-026-00763-7 (PMC13144423; doi:10.1038/s44318-026-00763-7)
Supplement: Supplementary file 4 — Source data Fig. 3 [file 44318_2026_763_MOESM4_ESM.zip › Source data Fig. 3.pptx]

## Slide 1
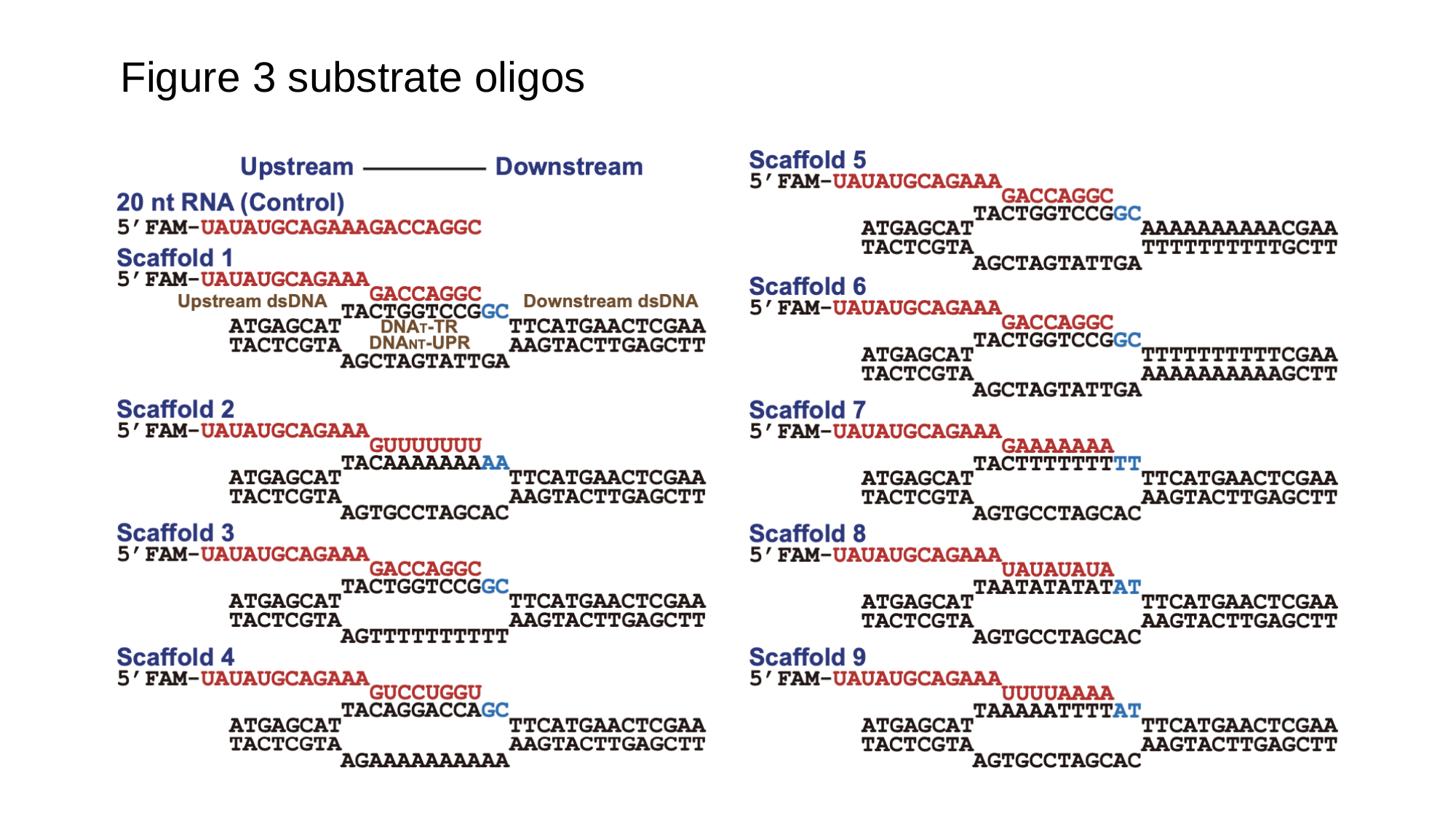

Figure 3 substrate oligos

## Slide 2
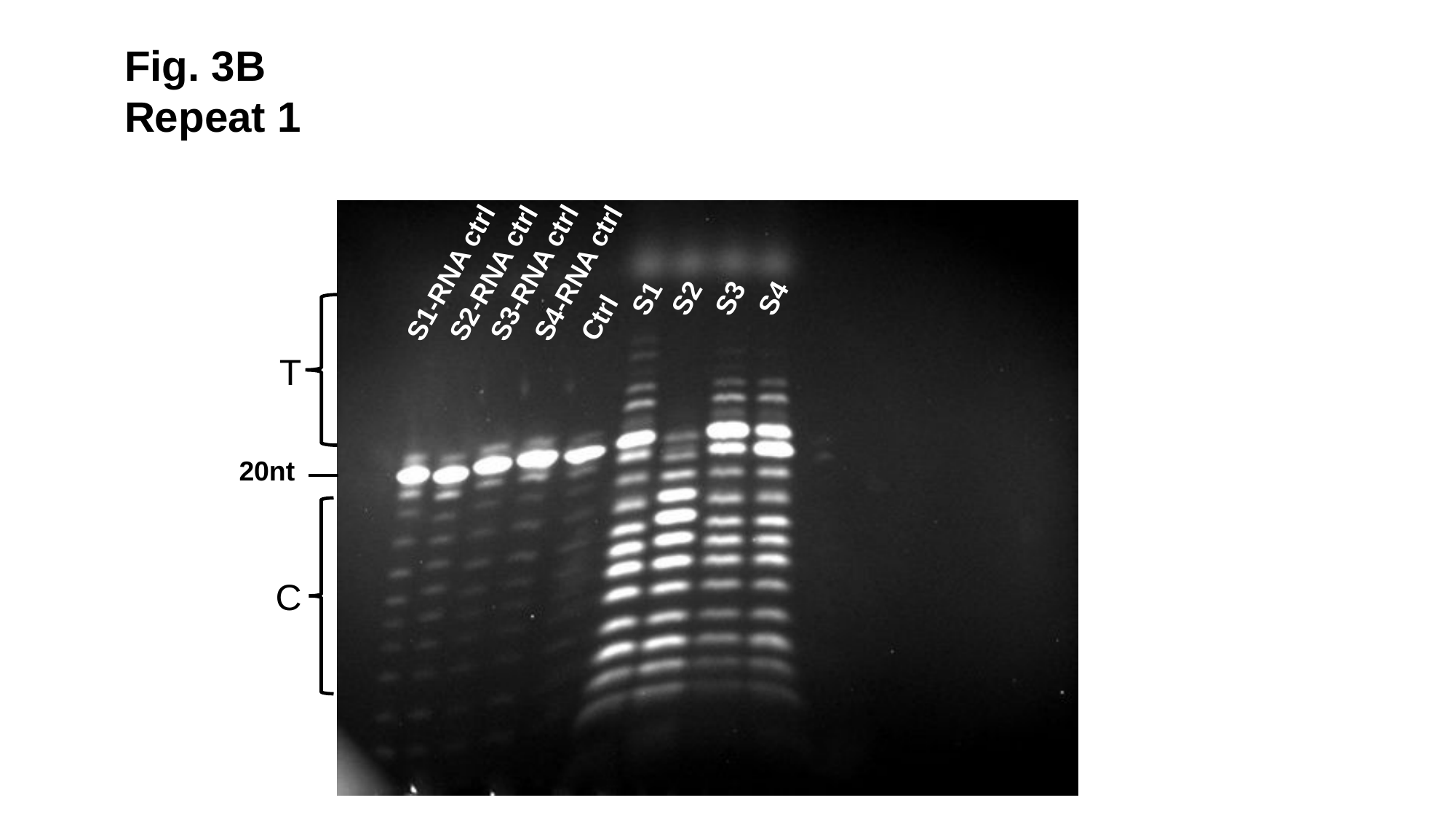

Fig. 3B
Repeat 1
S3-RNA ctrl
S1-RNA ctrl
S2-RNA ctrl
S4-RNA ctrl
S1
S2
S3
S4
Ctrl
T
20nt
C

## Slide 3
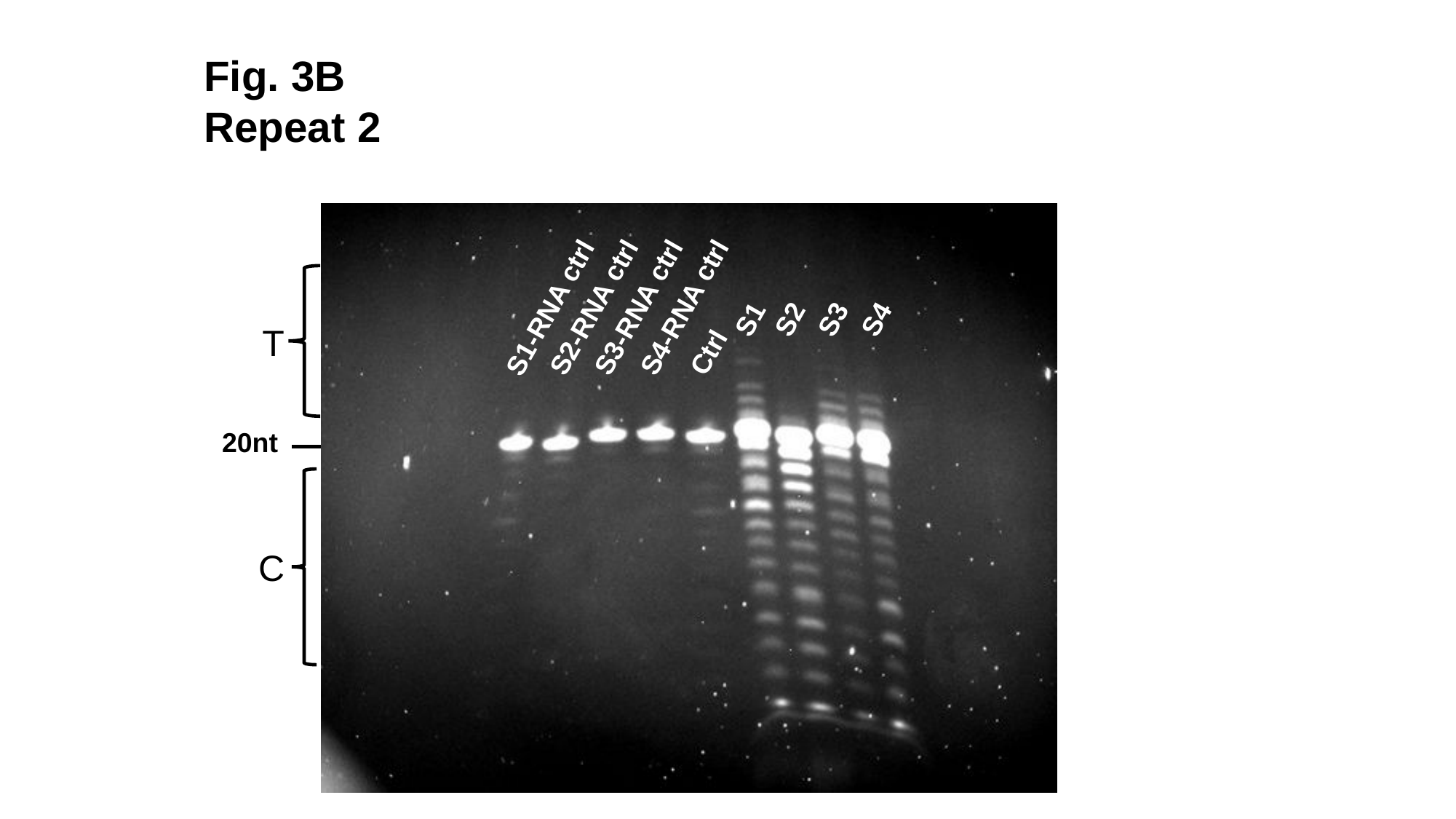

Fig. 3B
Repeat 2
T
20nt
C
S3-RNA ctrl
S1-RNA ctrl
S2-RNA ctrl
S4-RNA ctrl
S2
S3
S4
S1
Ctrl

## Slide 4
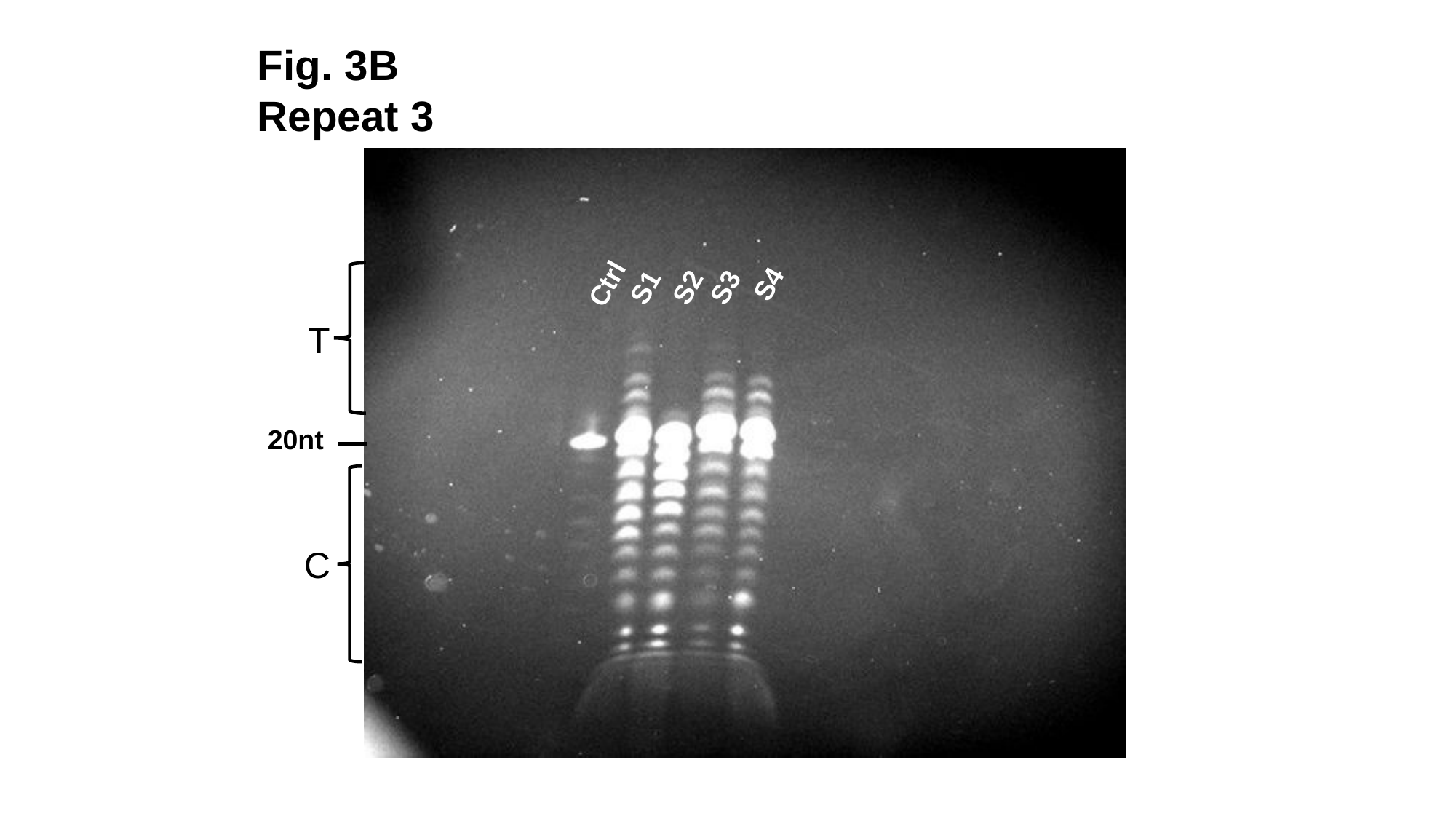

Fig. 3B
Repeat 3
Ctrl
S1
S2
S3
S4
T
20nt
C

## Slide 5
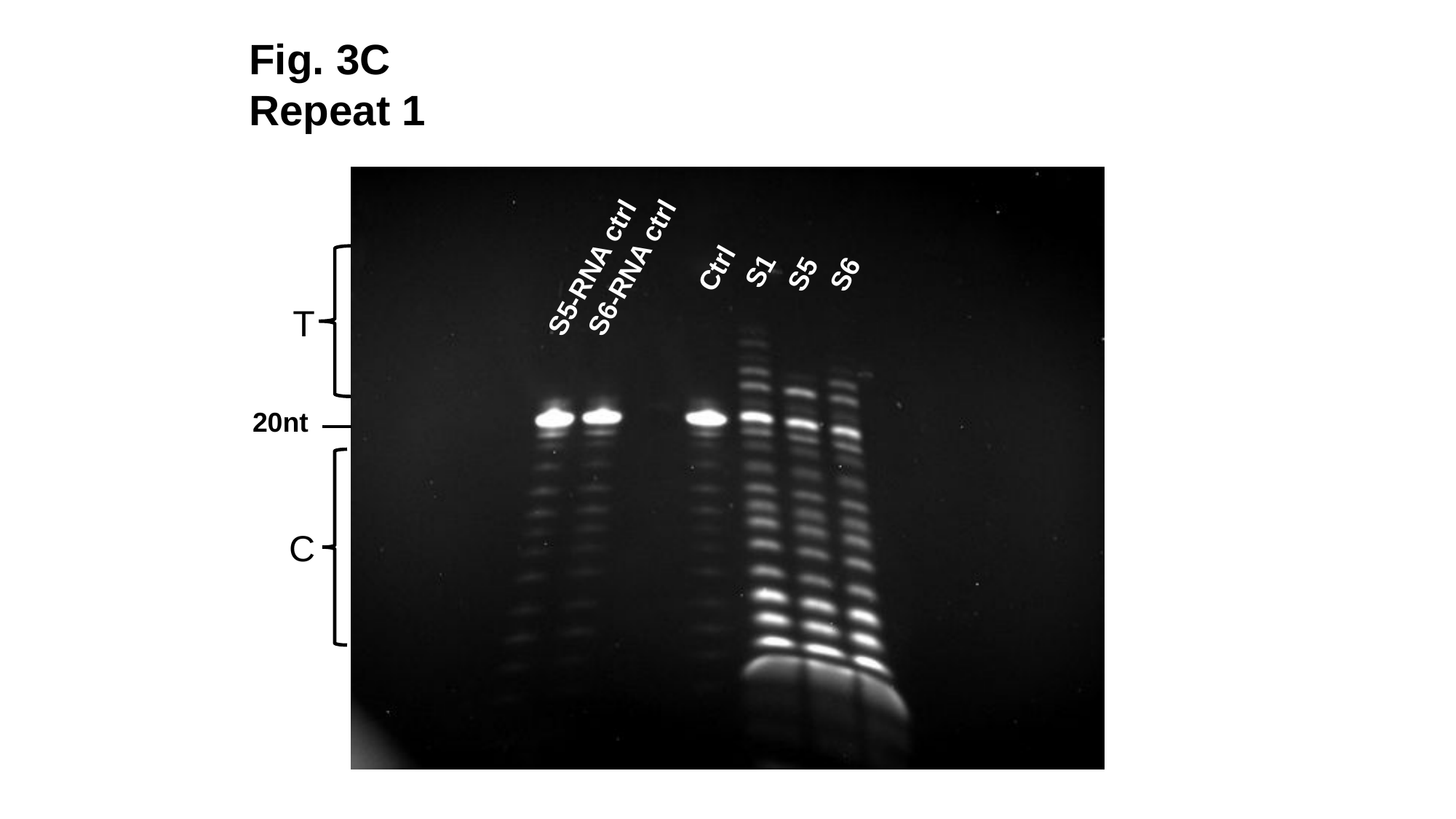

Fig. 3C
Repeat 1
Ctrl
S5-RNA ctrl
S6-RNA ctrl
S1
S6
S5
T
20nt
C

## Slide 6
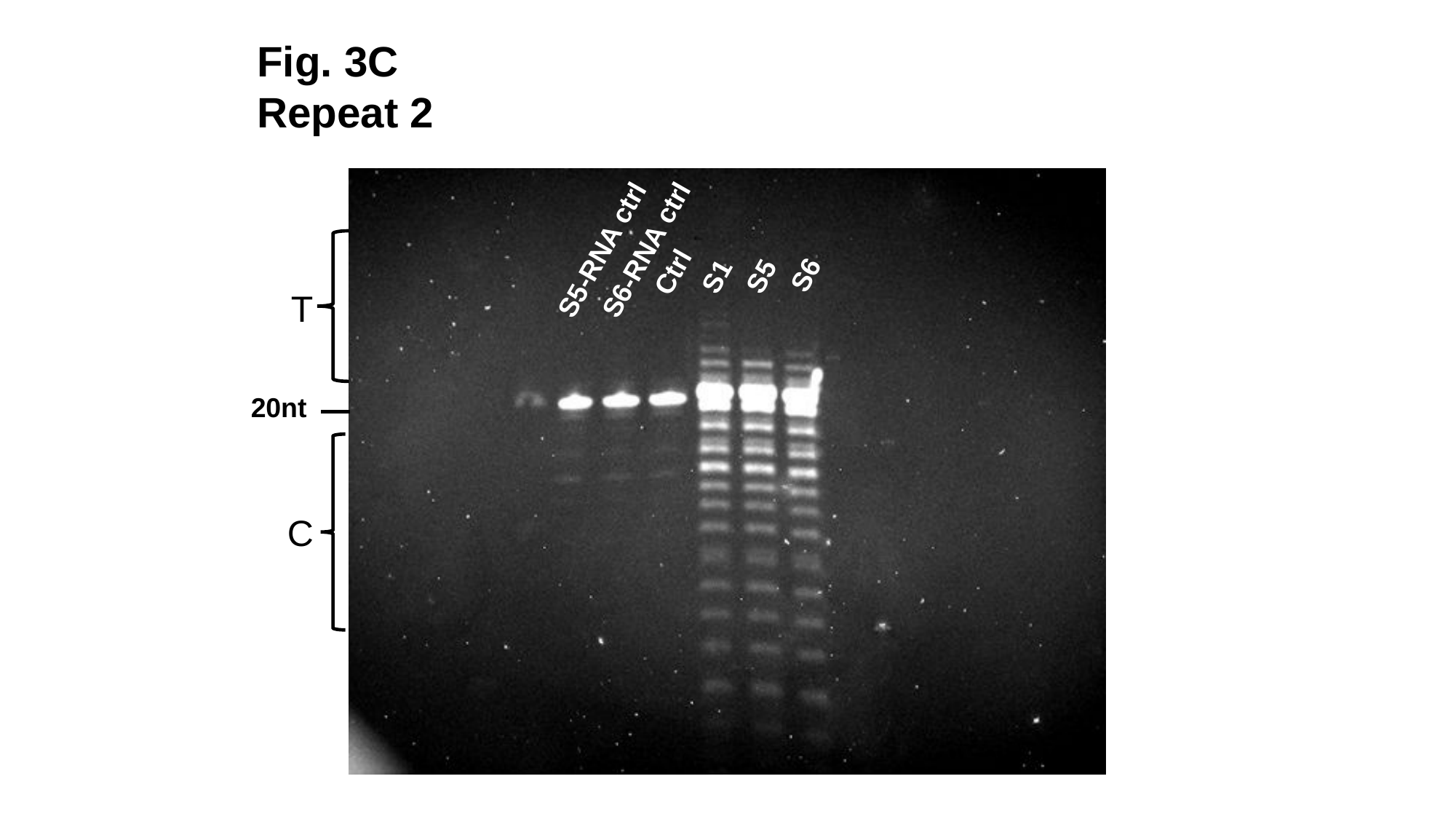

Fig. 3C
Repeat 2
S5-RNA ctrl
S6-RNA ctrl
Ctrl
T
20nt
C
S6
S1
S5

## Slide 7
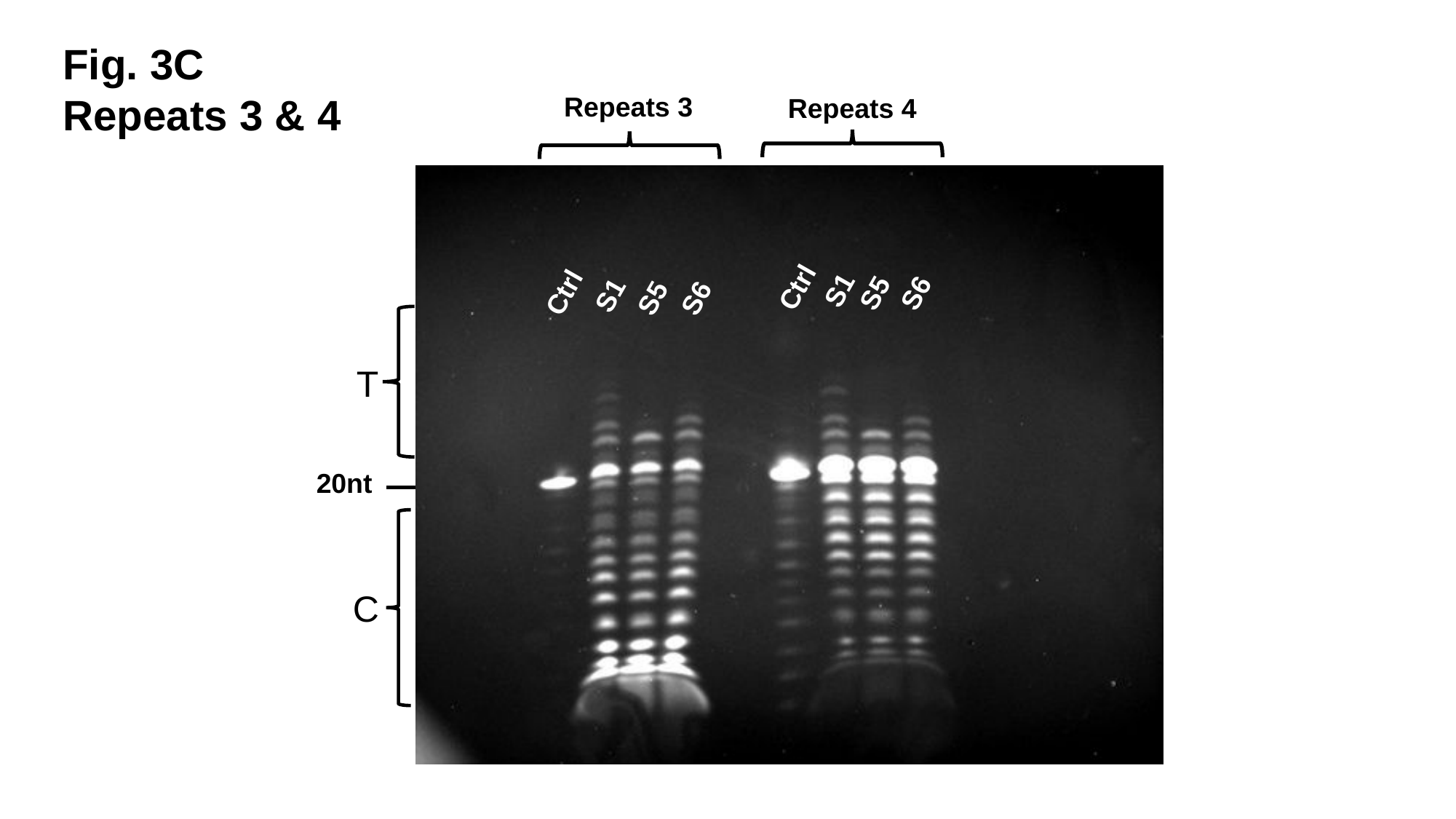

Fig. 3C
Repeats 3 & 4
Repeats 3
Repeats 4
Ctrl
Ctrl
S1
S6
S5
S1
S6
S5
T
20nt
C

## Slide 8
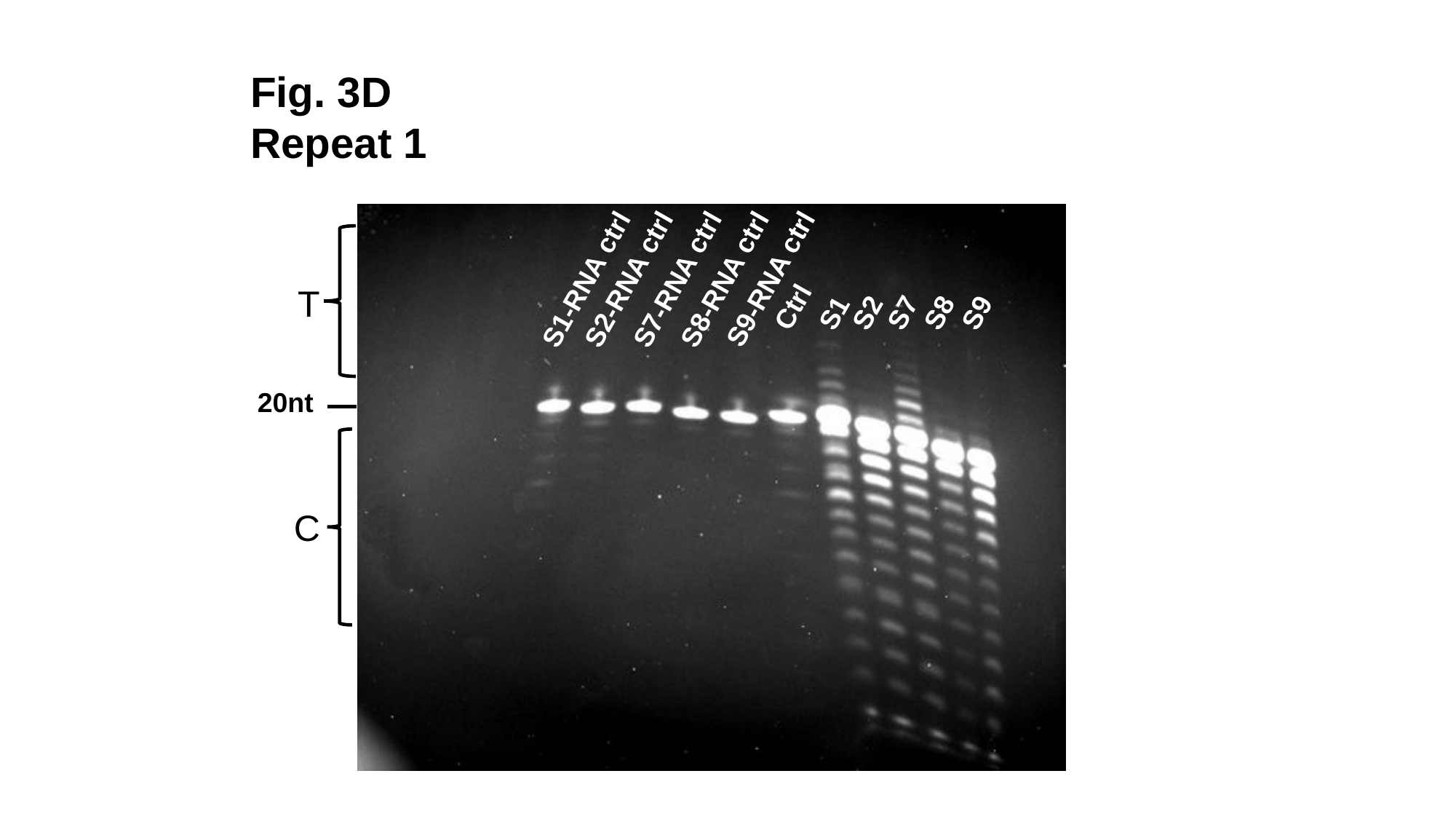

Fig. 3D
Repeat 1
T
20nt
C
S7-RNA ctrl
S1-RNA ctrl
S2-RNA ctrl
S9-RNA ctrl
S8-RNA ctrl
Ctrl
S2
S7
S8
S9
S1

## Slide 9
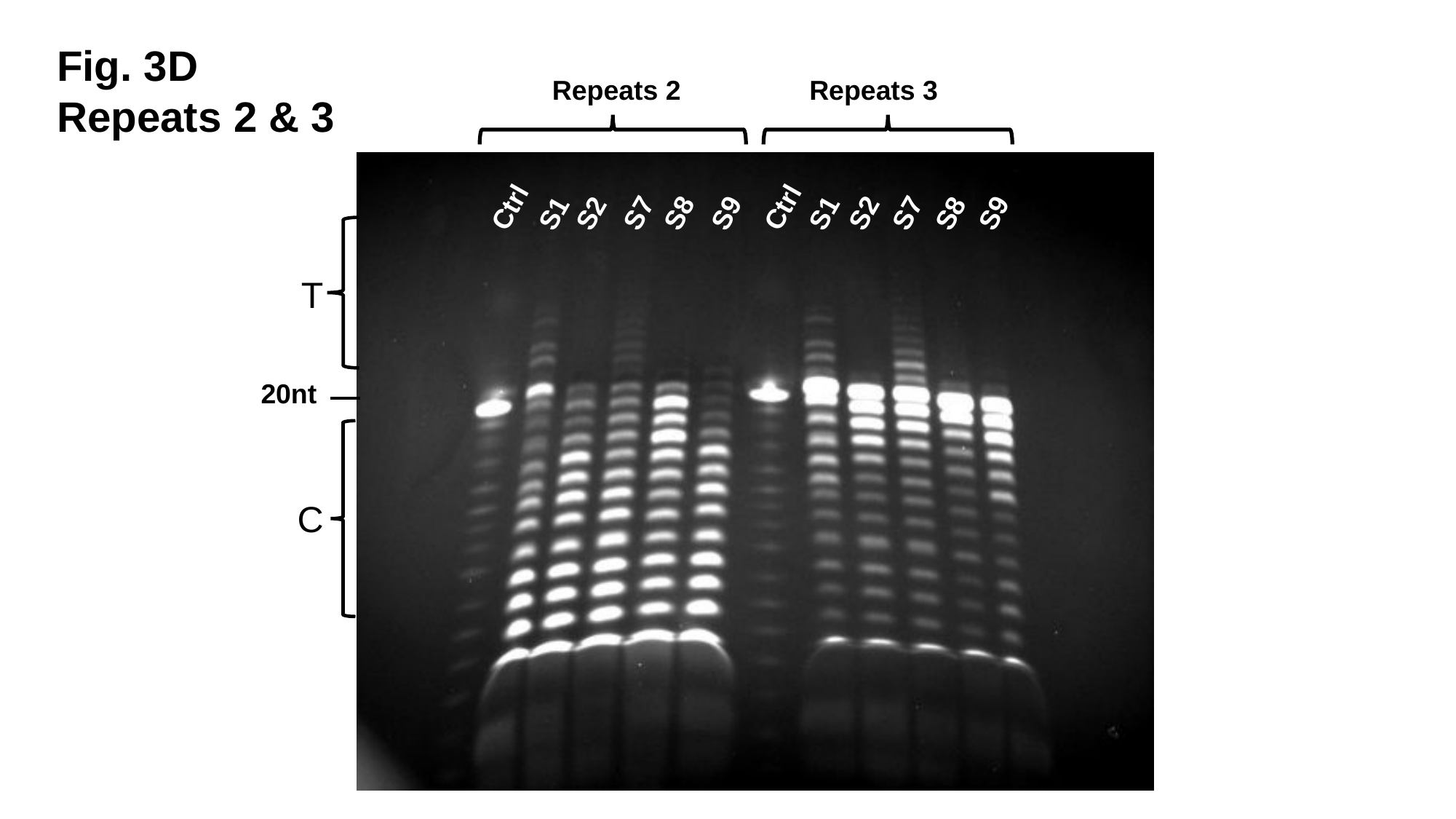

Fig. 3D
Repeats 2 & 3
Repeats 2
Repeats 3
Ctrl
Ctrl
S2
S7
S8
S2
S7
S8
S9
S9
S1
S1
T
20nt
C
